# Bioengineering approaches to dynamic impact analysis for cranial fracture interpretation in arcaheology

**DOI:** 10.1038/s41598-026-38313-0

**Published:** 2026-02-11

**Authors:** Daniel Rodríguez-Iglesias, Ana Pantoja-Pérez, Ángel De La Rosa, Pedro Latorre-Carmona, Nohemi Sala

**Affiliations:** 1 Centro Nacional de Investigación sobre Evolución Humana (CENIEH), Burgos, Spain; 2 Centro UCM-ISCIII de Evolución y Comportamiento Humanos, Avd/Monforte de Lemos, 5, Pabellón 14, Madrid, 28029 Spain; 3 Departamento de Tecnología Química, Energética y Mecánica, DIMME, Grupo de Durabilidad e Integridad Mecánica de Materiales Estructurales, Universidad Rey Juan Carlos, Madrid, Spain; 4 Departamento de Ingeniería Informática, Universidad de Burgos, Avda. Cantabria s/n, Burgos, 09006 Spain

**Keywords:** Fracture mechanics, Forensic anthropology, Trauma analysis, Interpersonal violence, Depressed fracture, Bone thickness, Anatomy, Medical research

## Abstract

Cranial fractures are widely documented in archaeological contexts, yet the application of fracture mechanics to differentiate traumatic events remains limited. This study analyses a dataset of 234 human cadavers subjected to 329 experimentally controlled blunt-impact tests, examining mechanical variables and fracture patterns that could be relevant to archaeological interpretation. The results show substantial methodological variability across the analysed studies. Analysis of these studies indicates that impact energy is the most reliable parameter for assessing fracture severity, suggesting a preliminary fracture threshold of around 2000 N, and that bone thickness is a major determinant of cranial resistance. Clear differences in fracture morphology according to impact surface were also observed: focal surfaces frequently produce depressed and comminuted fractures, whereas broad surfaces predominantly generate linear fractures. These data provide a framework for archaeological analysis: bone thickness, fracture morphology, and the presence and distribution of secondary fractures offer indirect but informative proxies for impact energy and surface characteristics, which could help to distinguish violent from non-violent events. This study emphasizes the need for dynamic fracture-mechanics approaches and targeted experimental work to better characterise archaeological impacts.

**Supplementary Information:**

The online version contains supplementary material available at 10.1038/s41598-026-38313-0.

## Introduction

Interpersonal violence constitutes a recurrent aspect of human behaviour and is widely documented in the archaeological record. The earliest well-documented case of interpersonal violence comes from the Middle Pleistocene site of Sima de los Huesos in Atapuerca (Spain). At this site, dated to around 430,000 years ago^1^ nine cranial individuals exhibit perimortem injuries consistent with interpersonal violence^2–4^. Among them, Cranium 17 stands out, having sustained two localized blows to the frontal squama delivered with the same object^2^. Although Cranium 17 remains the earliest case, other Pleistocene individuals have been proposed as possible examples of interpersonal violence, although their interpretation is more debated^5–10^. Further evidence from Holocene populations, including both mass graves^11,12^ and isolated cases^13^, demonstrates that cranial trauma linked to interpersonal violence persisted across diverse cultural and temporal contexts, underscoring the long-term continuity and variability of these behaviours throughout human history^14–18^.

Cranial fractures are one of the most frequently documented marks on the human skeleton in relation to episodes of interpersonal violence found in the record^11,12,16,18,19^. These fractures are typically associated with blunt and sharp force trauma because the cranium constitutes a preferential target when someone’s intention is to kill another individual due to the lethality of a blow delivered with sufficient force and speed^20–22^. In many cases, the accurate interpretation of these cranial lesions may be the only means of determining the cause and manner of an individual’s death^23^. This underscores the vital importance of examining cranial fractures in the context of interpersonal violence research.

Fracture pattern analysis is a crucial component of taphonomy^22,24,25^. Current analyses of cranial injuries include the evaluation of antemortem, perimortem fractures and postmortem alterations, as well as the biomechanics of cranial fractures, in order to determine the associated bioengineering variables (force, energy, or velocity)^25–27^. This holistic approach encompasses the identification, description, and interpretation of trauma patterns, associating them with interpersonal violence when applicable, and potentially linking these traumas to specific objects.

The archaeological context provides limited opportunities for studying the mechanics of cranial fractures and for quantifying the variables involved in interpersonal violence events. The few existing experimental studies often rely on different human analogues and are primarily focused on comparing fracture morphologies^28–30^.

In this context, forensic science and bioengineering can offer significant insights into the origins of archaeological head injuries. Extensive experimental research has been conducted to enhance our understanding of fracture mechanisms in the human head and to develop tolerance criteria for different parts of the cranium. Experimental studies on human specimens have substantially advanced knowledge of bone fracture mechanics and trauma^24,25,29–45^. By applying existing forensic and bioengineering data to the archaeological record, we can better delineate the mechanics of cranial fractures and identify key variables associated with their formation. Although physical impact parameters cannot be measured directly on archaeological material, integrating biomechanical knowledge with archaeological observations could provide a more explicit framework for evaluating the mechanical conditions that generated traumatic events in the past.

Another major challenge to the analysis of cranial fractures is the lack of uniform criteria for their characterisation in the archaeological fields. Research tends to use different approaches to describe and classify fractures, thus impeding proper comparison of results to in turn coherently interpret the data. This problem is exacerbated by the methodological diversity found in related disciplines, such as forensics and medicine, which use different analytical criteria based on their specific objectives. For example, in the medical field, fracture analysis often includes the assessment of associated soft tissues, allowing for the identification of compound fractures and other specific categories that are not always applicable to studies of the past^31,32^. This methodological heterogeneity complicates fracture analysis in the archaeological record.

The primary objective of this study is to analyse biomechanical data generated by bioengineering human modern experimental tests and evaluate their applicability to archaeological contexts. By examining how the structural characteristics of the cranium respond to different types of energies and impact surfaces, this research aims to develop a more accurate understanding of fracturing dynamics. A key focus is exploring the correlation between cranial fracture characteristics and the properties of impact surfaces, including variables like the total area of the objects involved. This approach establishes an initial systematic framework to link fracture morphologies to the types of surfaces that generate them, offering new insights into the interpretation of potential violent events in the past.

Additionally, this study reviews and compares fracture criteria used in forensic science and medicine, with the aim of proposing a unified system adapted to the archaeological record. By integrating these perspectives, this research seeks to provide a consistent and precise methodology that enhances the interpretation of fracture mechanics and contributes to the development of more robust analytical tools in this field.

## Results

### Database built

The final database includes a total of 234 cadavers subjected to 329 impacts. Of these, 172 impacts were performed on whole human bodies (69 unembalmed and 103 embalmed), while the remaining impacts were conducted on isolated human heads, using 157 heads (103 unembalmed and 54 embalmed).

The number of impacts exceeds the number of samples because some studies used the same subject to perform multiple impacts on different regions of the cranial vault. The most frequently impacted cranial regions were the parietal (131 impacts), followed by the frontal (97 impacts) and the temporo-parietal (84 impacts) regions. The occipital region received the fewest impacts, with only 17 impacts recorded. The predominant experimental setups included drop towers and pneumatic impactors, although free-fall and pendulum tests were also documented.

A total of 236 fracture tests were documented in the sample, while 33 cases did not exhibit any signs of fracture. The impact forces used ranged from 1,340 N to 17,000 N across 275 impacts. Absorbed energy varied from 8.98 J to 152.7 J in 152 recorded impacts, while loading velocities ranged from 1.58 m/s to 8.14 m/s for 270 impacts (Table S1 and Table S2). It is important to note that no fractures were observed in impacts performed with forces below 2,000 N as also reported Cormier et al.^33^. This value could be considered a preliminary threshold for fracturing, provided the variability between samples and the specific conditions of the experiments are taken into account.

Finally, although the experiments conducted by Hodgson^34–36^ were included in the dataset, they were excluded from statistical analyses due to identified errors in the calculation of loading velocities. Given the potential for additional inconsistencies in the reported values, these data were omitted from the main analyses. The full dataset is provided as Supplementary Data 1 (Excel file).

## Effect of physical variables on mechanical response

### Absorbed energy vs. peak force

The relationship between absorbed energy and maximum force plays a crucial role in the analysis of documented fractures. An object’s energy is dependent on its momentum and mass, and changes in energy are the result of forces acting on the object^37,38^. These forces are integral to understanding how energy is transferred and how force can vary in response to changes in energy, which can affect fracture behavior.

**Fig. 1:**
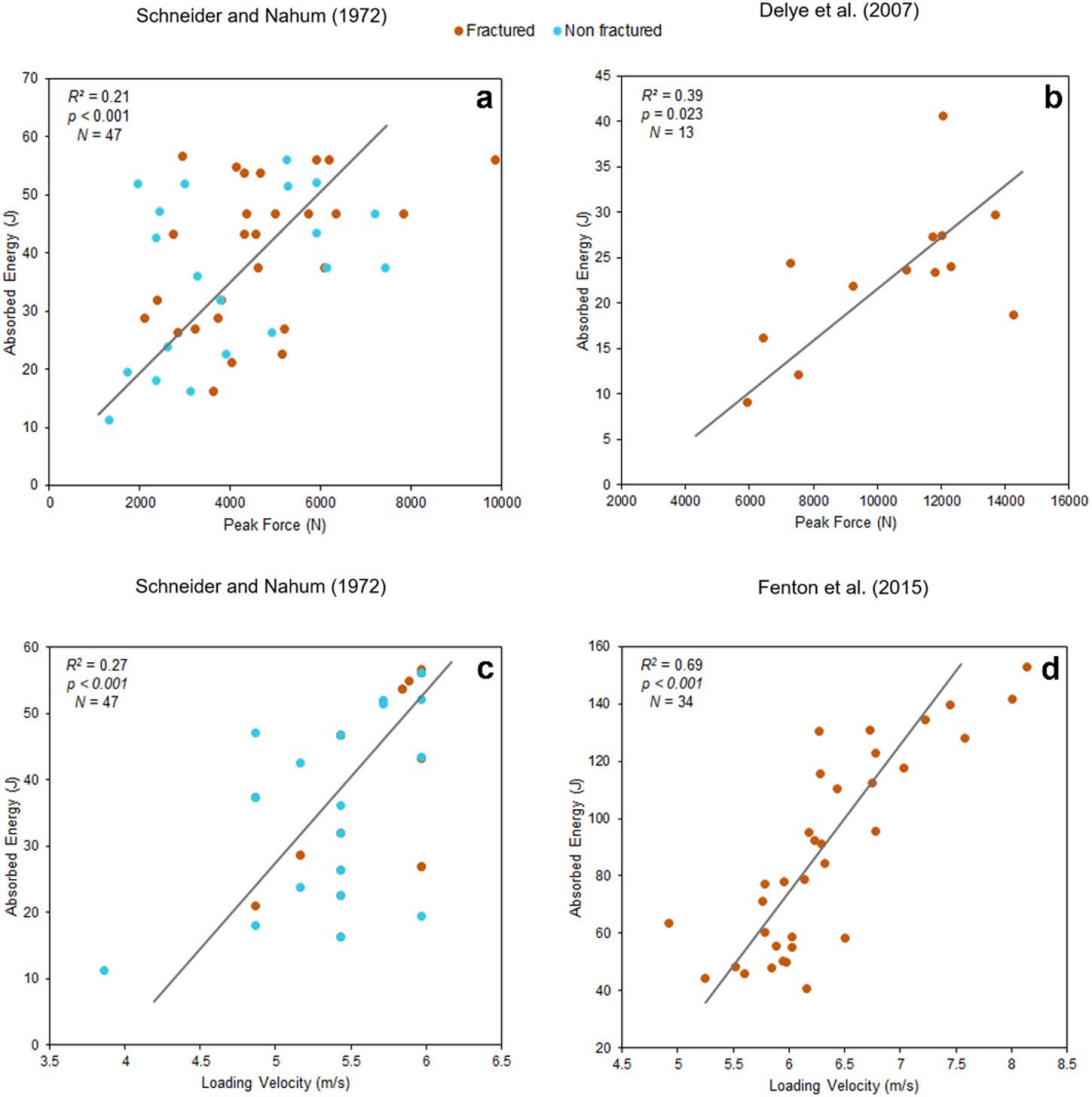
**a** and **b**) Correlation plots between absorbed energy and peak force. **c** and **d**) Correlation plots between absorbed energy and loading velocity. Studies carried out by Schneider and Nahum^39^using a drop tower set up, Delye, et al^40^. using a pendulum set up and Fenton, et al^41^., published by Isa, et al^26,27^., using a pneumatic system. The plots were generated using the data provided in the papers.

Using data from Schneider and Nahum^39^, we identified a statistically significant but weak correlation between absorbed energy and peak force (*R²* = 0.21, *p* < 0.001, *N*= 47). The considerable scatter in the data supports this finding. Data from Delye, et al^40^. revealed a moderate but significant correlation between absorbed energy and peak force (*R²* = 0.39, *p* = 0.023, *N* = 13) (Fig. 1). Similarly, analysis of data from Fenton, et al^41^., and reported by Isa, et al^26,27^., showed a statistically significant but weaker correlation (*R²* = 0.27, *p* < 0.001, *N*= 34). In contrast, analysis of data from Yoganandan, et al^42^., obtained using a pneumatic impactor, revealed no significant correlation (*R²* = 0.0 6, *p* = 0.635, *N* = 6), and the wide dispersion of data points further reinforces the absence of a clear trend (Figure S1).

### Absorbed energy vs. loading velocity

The correlation between absorbed energy and loading velocity is a key factor in understanding impact dynamics, as the relationship between the two of is quadratic nature. According to the principle of kinetic energy, the energy absorbed during impact increases with the square of the velocity, meaning that even small increases in velocity can lead to a significant rise in absorbed energy^37,38^. This quadratic nature of the relationship makes it particularly important in impact analysis^43,44^.

The correlation between absorbed energy and loading velocity in the study by Schneider and Nahum^39^ shows a positive and statistically significant relationship (*R²* = 0.27, *p* < 0.001, *N* = 47). Notably, the data dispersion was lower compared to the correlation values between peak force and absorbed energy, suggesting that this relationship is more consistent and less influenced by external factors. The strongest correlation was found in the study by Fenton, et al. 36, published by Isa, et al. 15,16, where loading velocity and absorbed energy showed a positive and highly significant relationship (R² = 0.69, *p* < 0.001, *N* = 34) (Fig. 1).

However, in the study carried out by Yoganandan, et al^42^. (*R²* = 0.29, *p* = 0.274, *N*= 6) and Delye, et al^40^. (*R²* = 0.05, *p* = 0.451, *N* = 13), the relationship was not statistically significant. Additionally, the high data dispersion around the trend line further emphasizes the lack of a consistent correlation between these variables (Figure S2).

### Peak force vs. loading velocity

The correlation between peak force and loading velocity is a key factor in impact dynamics, since higher loading velocities typically result in greater peak forces due to the need to modify the momentum over a shorter time period. This aligns with Newton’s Second Law of Motion, which states that force is equal to the rate of change of momentum. Consequently, as the loading velocity increases and the impact time decreases, the peak force generated tends to be higher^37,38^. However, this relationship is not consistently observed across all studies.

**Fig. 2:**
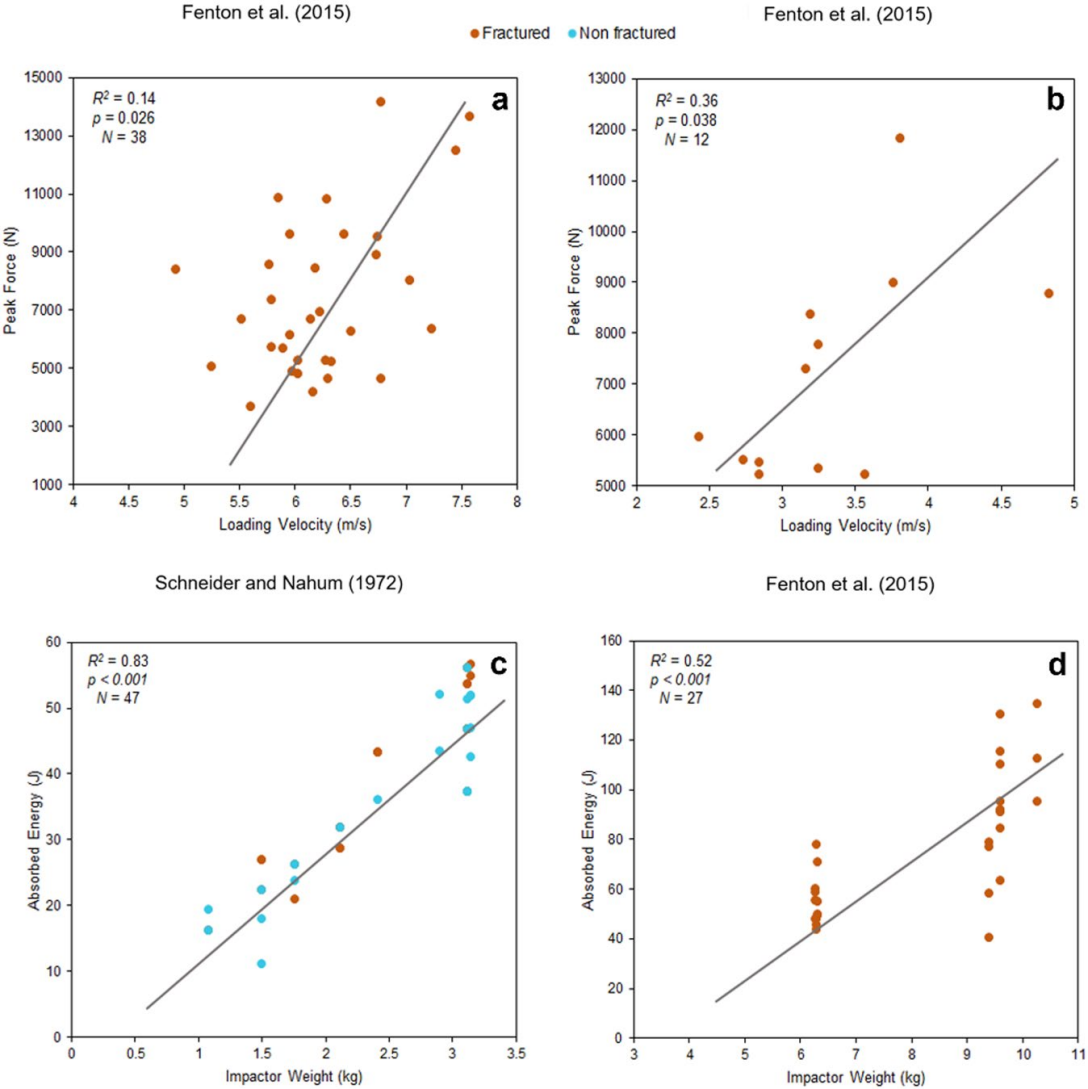
**a** and **b**) Correlation plots between peak force and loading velocity. **c** and **d**) Correlation plots between absorbed energy and impactor weight. Studies carried out by Schneider and Nahum^39^and Fenton, et al^41^. using a drop tower set up. Fenton, et al^41^., published by Isa, et al^26,27^., also using a pneumatic system (a and d). The plots were generated using the data provided in the papers.

In the studies conducted by by Fenton, et al^41^. and published by Isa, et al^26,27^., using a pneumatic impactor (*R²* = 0.14, *p* = 0.026, *N* = 38) and a drop tower (*R²* = 0.36, *p* = 0.038, *N* = 12) they demonstrate a positive, albeit weak, correlation. In both cases, the relationships are statistically significant but of moderate magnitude (Fig. 2).

On the other hand the analysis of the studies conducted by Schneider and Nahum^39^ (*R²* = 0.05, *p* = 0.144, *N*= 47),Yoganandan, et al^42^. (*R²* = 0.23, *p* = 0.34, *N*= 6), and Delye, et al^40^. (*R²* = 0.04, *p* = 0.53, *N* = 13), no statistically significant relationship was found between peak force and loading velocity. The wide dispersion of the data further highlights the weak correlation between these variables (Figure S3 and S4).

### Absorbed energy vs. impactor weight

The force exerted by an object is proportional to its mass and acceleration. Therefore, a heavier impactor generally transfers more energy to the cranium (French 2024; Greenwood 1988). Accordingly, understanding the relationship between absorbed energy and impactor weight is essential to assessing how mass affects energy dissipation during impact, whether through release or material failure.

Data from Schneider and Nahum^39^ indicate a strong correlation between absorbed energy and impactor weight, with *p* < 0.001 and a coefficient of determination *R*^*2*^ = 0.83 (*N*= 47). Similarly, the results from Fenton, et al^41^. and Isa, et al^26,27^. are also statistically significant, with *p* < 0.001 and an *R*^*2*^ = 0.52 (*N* = 27) (Fig. 2).

### Peak force vs. impactor weight

Impactor weight and peak force offer valuable insight into how mass influences peak force generation during an impact. The magnitude of the force can vary depending on the impactor’s weight, the deceleration it undergoes upon collision, and the structural resistance of the impacted material. Force is the rate of change of momentum. Therefore, as the weight of the impactor increases, the peak force generated during the collision also increases, assuming the velocity and deceleration are constant^37,38^.

**Fig. 3:**
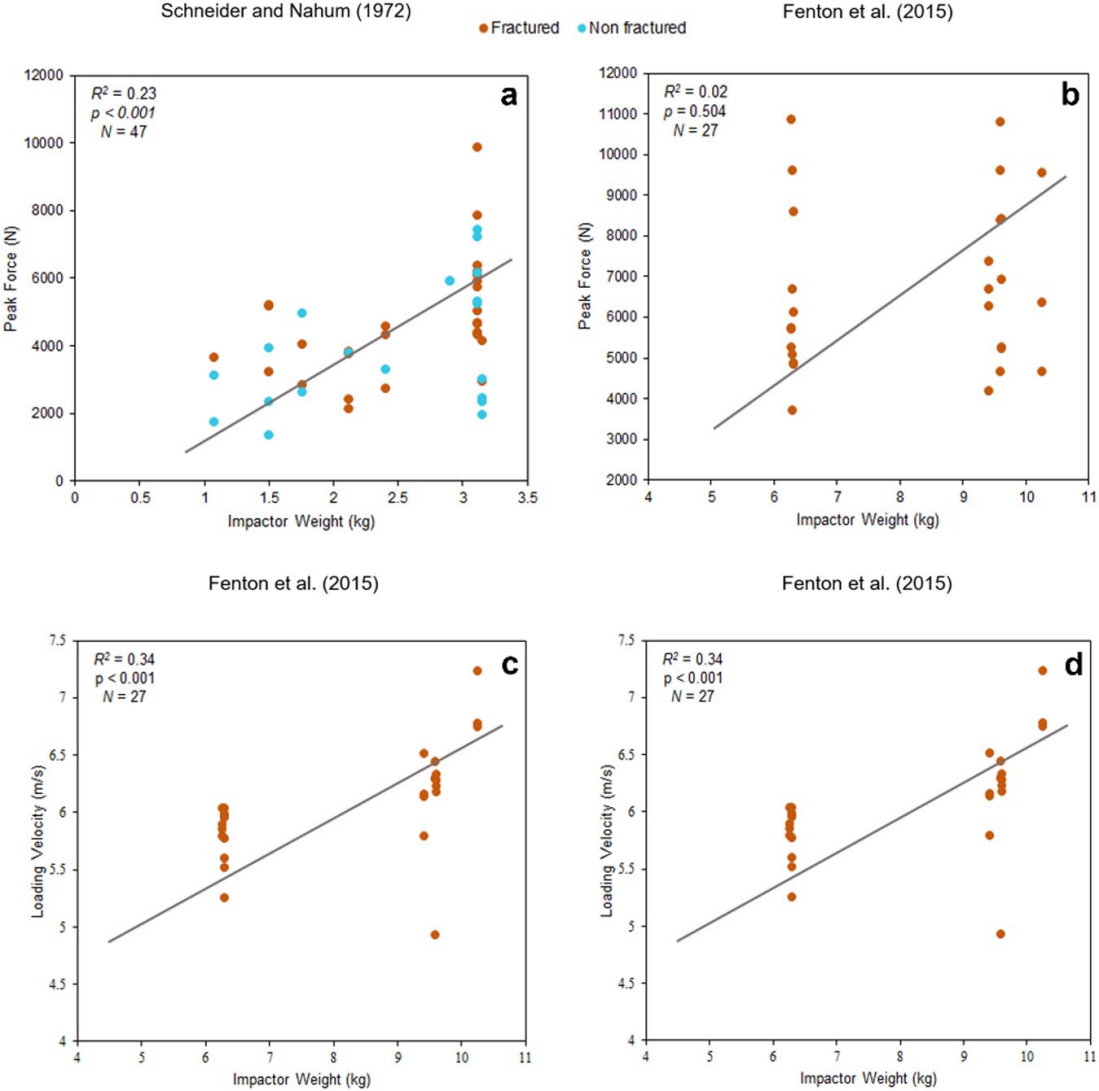
**a** and **b**) Correlation plots between peak force and impactor weight. **c** and **d**) Correlation plots between loading velocity and impactor weight. Studies carried out by Schneider and Nahum^39^and Fenton, et al^41^. using a drop tower set up. Fenton, et al^41^., published by Isa, et al^26,27^., also using a pneumatic system (b and c). The plots were generated using the data provided in the papers.

In the case of Schneider and Nahum^39^, the impactor’s weight exhibits a significant influence on peak force (*N* = 47), with a coefficient of determination of *R*^*2*^ = 0.23 and a statistically significant *p*< 0.001. However, the analysis of studies conducted by Fenton, et al^41^. and published by Isa, et al^26,27^. using a pneumatic impactor (*R*^*2*^ = 0.02, *p* = 0.504, *N* = 27) and a study with a drop tower (*R*^*2*^ = 0.12, *p* = 0.261, *N* = 12) did not reveal statistically significant relationships (Fig. 3 and Figure S5).

### Loading velocity vs. impactor weight

Loading velocity and impactor weight are key variables in understanding impact dynamics, as they jointly determine the energy transferred during a collision. Since force is the rate of change of momentum, the impactor’s mass contributes inertia and momentum, while velocity influences both momentum and kinetic energy. Thus, greater mass and velocity result in higher momentum and energy, which in turn affect the severity and outcome of the impact (French 2024; Greenwood 1988).

Analysis of the Schneider and Nahum^39^ data revealed no significant correlation between loading velocity and impactor weight (*N* = 47), as indicated by *p* = 0.268 and *R*^*2*^= 0.03 (Figure S6). In contrast, a positive correlation was observed in the studies conducted by Fenton, et al^41^. and published by Isa, et al^26,27^., using a pneumatic impactor (*R*^*2*^ = 0.34, *p* < 0.001, *N* = 27) and a drop tower (*R*^*2*^ = 0.37, *p* = 0.0356, *N* = 12) (Fig. 3).

### Comparative analysis of physical variables across studies

When comparing data from different studies, consistent correlations were only obtained when analysing experimental test from Schneider and Nahum^39^, Fenton, et al^41^., Isa, et al^15,16^. together. This suggest that the experimental methods and testing conditions used in each study may be influencing the observed relationships in different ways. A summary of all correlations can be found in the supplementary material (Table S3).

**Fig. 4:**
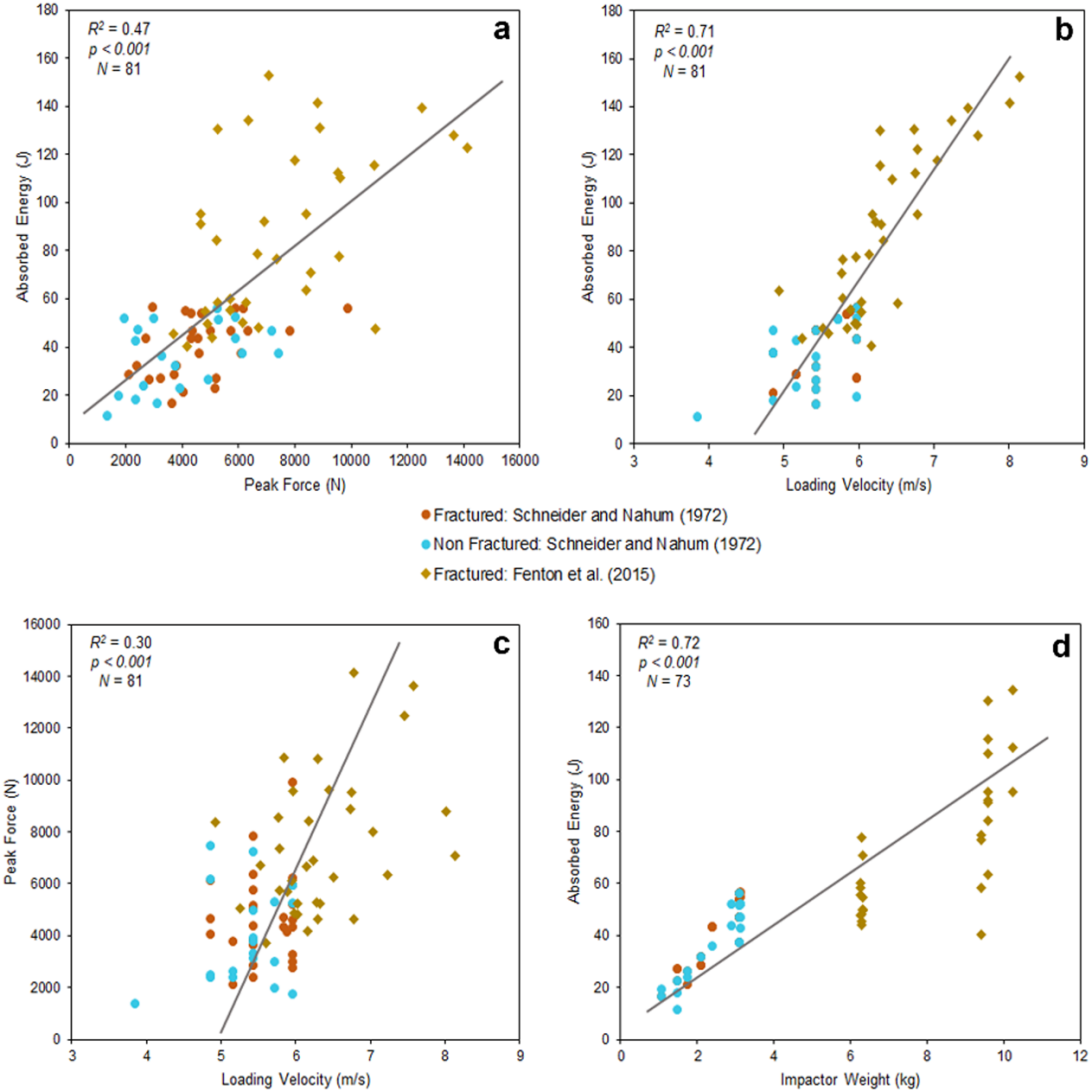
Comparative analysis of data from Schneider and Nahum^39^using a drop tower set up and Fenton, et al^41^., published by Isa, et al^26,27^., using a pneumatic system. **(a)** Correlation plot between absorbed energy and peak force. **(b)** Correlation plot between absorbed energy and loading velocity. **(c)** Correlation plot between peak force and loading velocity. **(d)** Correlation plot between absorbed energy and impactor weight. The plots were generated using the data provided in the papers.

The relationship between absorbed energy vs. peak force (*R*^*2*^ = 0.47, *p* < 0.001, *N* = 81), absorbed energy vs. loading velocity (*R²* = 0.71, *p* < 0.001, *N* = 81) and peak force vs. loading velocity (*R²* = 0.30, *p* < 0.001, *N* = 81) show a positive correlation, with a high statistical significance. The data align consistently along the regression line for absorbed energy and loading velocity, suggesting a strong and stable relationship between these two variables, while energy and peak force and peak force and loading velocity shows dispersion of the points (Fig. 4).

On the other hand, absorbed energy vs. impactor weight (*R*^*2*^ = 0.72, *p* < 0.001, *N* = 73), impactor weight vs. peak force (*R*^*2*^ = 0.31, *p* < 0.001, *N* = 73) and impactor weight vs. loading velocity (*R*^*2*^ = 0.37, *p* < 0.001, *N* = 73) shows also a positive correlation and highly statistically significant relationship. In spite of this, it is worth noting how the data tends to be grouped together by study in the graph (Fig. 4 and Figure S7).

## Effect of anatomical variables on mechanical response

Traditionally, both bone thickness and soft tissues have been considered critical factors in the energy absorption capacity of the cranium during an impact^45–47^. Furthermore, it has been proposed that hair, particularly in individuals with thick or bushy hair, may influence the distribution of impact forces, potentially playing a significant role in the likelihood and pattern of fractures in the human cranial vault^36^. However, the exact influence of soft tissues remains unclear.

**Fig. 5:**
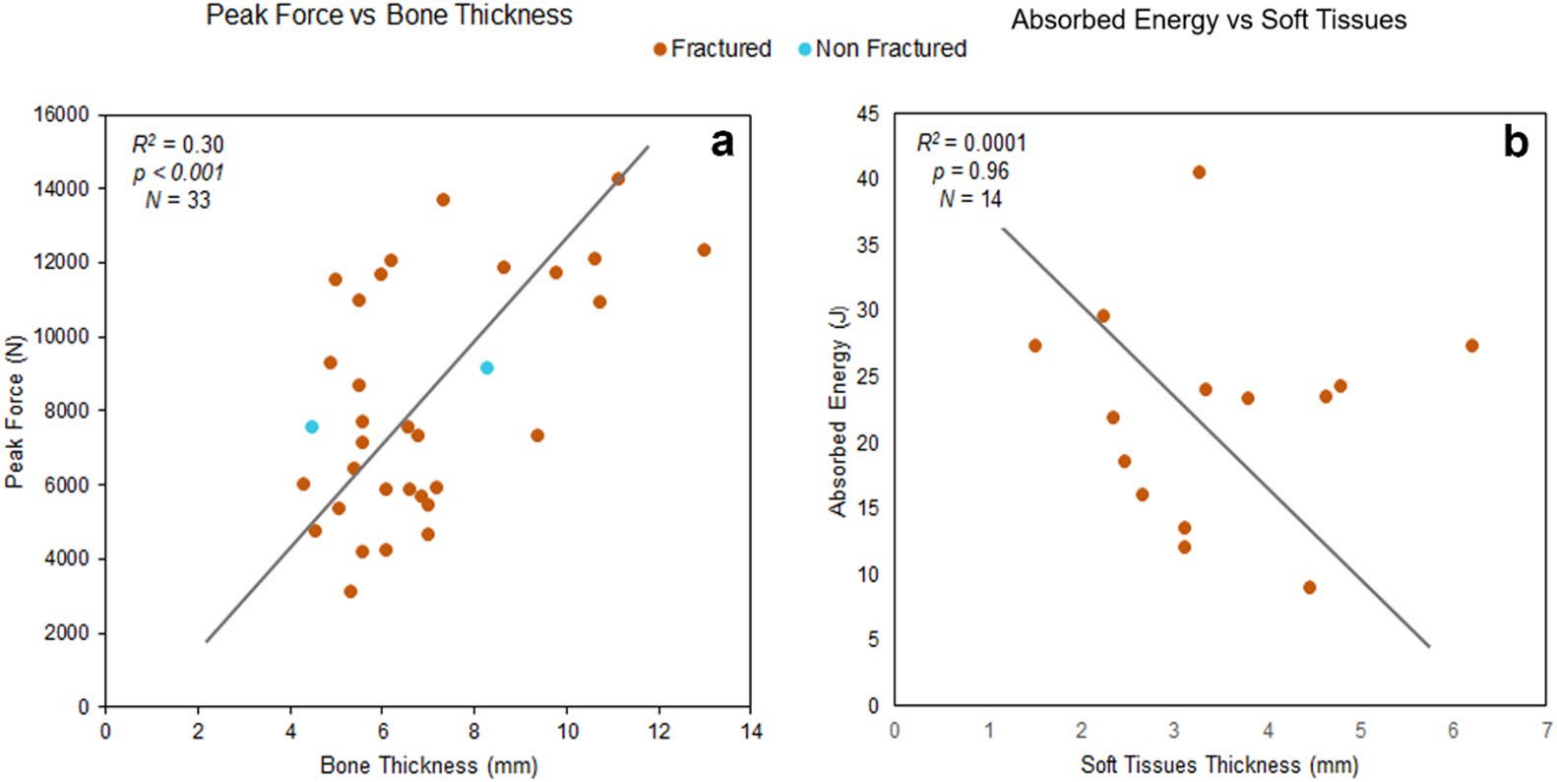
Correlations between physical impact variables and anatomical variables. **(a)** Correlation between peak force and bone thickness. **(b)** Correlation between peak force and soft tissues. The plots were generated using the data provided in the papers.

Bone thickness and peak force were significantly positively correlated (*R²* = 0.30, *p* < 0.001, *N* = 33) supporting the hypothesis that bone plays a critical role in cranial vault resistance capacity. In contrast, the relationships between soft tissue thickness and peak force (*R²* = 0.042, *p* = 0.347, *N* = 33) and between soft tissue thickness and absorbed energy (*R²* = 0.061, *p* = 0.057, *N* = 60) were not statistically significant (Fig. 5 and Figure S8). The former showed a negative correlation, supporting the idea that soft tissues may not substantially influence resistance to force. The latter showed a positive correlation, which despite not reaching significance, suggests a potential trend that could become more apparent with larger sample sizes.

## Fracture evaluation

### Classification of cranial vault fractures

Cranial vault fractures are important both because they are indicative of the severity of the trauma and because, depending on their location, they may be associated with various injuries. These fractures can be classified in many ways: anatomically, in relation to the overlying wound, by their morphology, by their degree of displacement, etc.

**Fig. 6:**
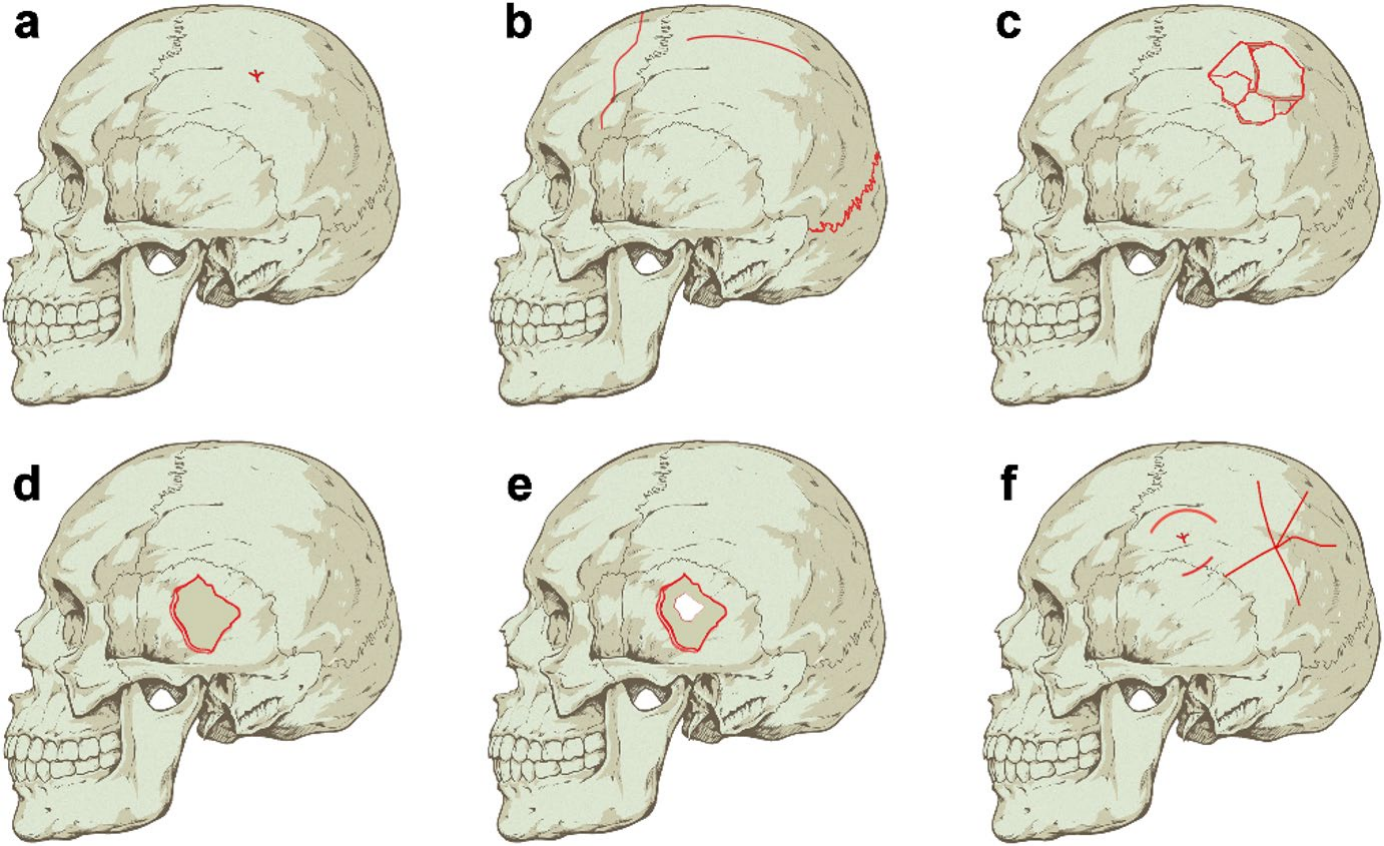
Morphology of cranial vault fractures. **a** Notch. **b** Linear fractures. **c** Depressed and comminuted fracture. **d** Penetrating fracture. **e** Perforating fracture. **f** Concentric and radial fractures. Illustrations were created by the corresponding author using Adobe Photoshop.

Our fracture analysis criteria were developed based on various classifications established in prior studies^3,22,26,27,31,32,39,46,48–54^ (Fig. 6 and Supplementary text 1). Cranial vault fractures were divided into two categories according to the time of occurrence for each fracture:

- **Primary fractures**: Primary fractures are those that occur immediately after trauma. They may manifest themselves at the site of impact or occur remotely, affecting another bone or site. These fractures have been classified into five basic types, distinguished by their morphology and the energy required to produce them: notch, linear, depressed, penetrating or perforating.
- **Non-primary fractures**: These fractures result from an initial impact but develop secondarily or tertiarily to primary fractures, manifesting around the impact site as a response to relieve excess pressure. This category of fractures includes radial, concentric, and comminuted fractures.

### Impact surface effects on fracture outcomes

A total of 122 impacts involving focal surfaces were analysed, using impactors with areas ranging from 5 to 6.45 cm². Additionally, 195 impacts with broad surfaces were studied, with areas between 15.7 and 1,295.13 cm², including free-fall tests where subjects impacted the ground. Twelve cases were excluded due to insufficient data on impactor dimensions, particularly in studies Kroman, et al^55^. and Hodgson and Thomas^36^.

Among the impacts conducted on focal surfaces, 24 tests were recorded that did not result in fractures, and 20 tests had descriptive criteria that were too ambiguous to be interpreted. In the remaining 78 tests, the following primary fracturing patterns were identified: 30 notches, 23 linear fractures, 24 depressed fractures, and one penetrating fracture. Additionally, associated secondary fractures were observed, including two radial fractures, three concentric fractures, and 27 comminuted fractures (Table 1).

**Table 1:** Distribution of primary and secondary fracture patterns depending on the impact surface used.

Surfaces	Primary fractures				Secondary fractures		
	Notch	Lineal	Depressed	Penetrating	Radial	Concentric	Comminuted
Focal	30 (38.46%)	23 (29.49%)	24 (30.77%)	1 (1.28%)	2 (6.25%)	3 (9.37%)	27 (84.37%)
Broad	1 (0.88%)	100 (88.5%)	12 (10.62%)	0 (0%)	8 (24.24%)	12 (36.36%)	13 (39.39%)

Regarding the impacts performed on broad surfaces, 11 tests were identified that did not result in fractures, while 71 tests which descriptive criteria were too ambiguous to be interpreted. Among the remaining 113 tests, the following primary fracture types were documented: one notch fracture, 100 linear fractures, and 12 depressed fractures. Additionally, associated secondary fractures were recorded, consisting of eight radial fractures, 12 concentric fractures, and 13 comminuted fractures (Table 1).

The focal surfaces were evenly distributed among the types of primary fracturing produced. This pattern suggests that impacts made with focal surfaces tend to produce primary diversified fractures. However, a high proportion of comminuted fractures was observed (84.37%). In contrast, impacts made on broad surfaces suggest a clear tendency toward linear fractures, which account for 88.5% of cases, while depressed fractures represent only 10.62%. The secondary fractures are more evenly distributed in this group, as opposed to the focal impact group, where the majority of secondary fractures are comminuted (84.37%).

The distribution of secondary fractures associated with primary fractures on focal surfaces shows that all three documented concentric fractures are linked to linear fractures, while 88.89% of comminuted fractures are associated with depressed fractures. In contrast, the association of secondary fractures with primary fractures on broad surfaces is evenly distributed across all secondary fracture types. Notably, no secondary fractures were documented in association with notches, as this type of fracture represents the minimal force required to fracture the cranial vault (Table 2).

**Table 2:** Distribution of secondary fractures associated with primary fractures as a function of the impact surface used. a) there are 13 comminuted fractures documented for the wide surfaces. Only 12 appear here because one of them is associated with a primary fracture whose ambiguous description did not allow for its interpretation.

Focal Surface				Broad Surface			
Primary fractures	Secondary fractures			Primary fractures	Secondary fractures		
	Radial	Concentric	Conminuted		Radial	Concentric	Conminuted
Notch	0 (0%)	0 (0%)	0 (0%)	Notch	0 (0%)	0 (0%)	0 (0%)
Lineal	0 (0%)	3 (100%)	4 (14.81%)	Lineal	5 (62.5%)	9 (75%)	7 (58.33%)
Depressed	1 (50%)	0 (0%)	22 (88.89%)	Depressed	3 (37.5%)	3 (25%)	5 (41.67%)
Penetrating	1 (50%)	0 (0%)	1 (3.70%)	Penetrating	-	-	-

## Discussion

The correlations between peak force and loading velocity are inconsistent and often statistically weak (Fig. 3). This pattern arises because peak force reflects the structural resistance of the cranial vault at the moment of failure, rather than the maximum force associated with the applied loading conditions. When a fracture occurs, the measurement captures the vault’s failure threshold, not the full magnitude of the impact. Consequently, peak force does not reliably represent the mechanical behaviour of variables such as loading velocity or absorbed energy.

In contrast, the correlations involving absorbed energy are more consistent because energy is directly dependent on impact velocity and, to a lesser extent, on peak force. Since energy integrates both the magnitude of loading and the dynamic conditions of the impact, it provides a more coherent parameter from a fracture mechanics perspective.

Energy absorption also varies with factors such as impact angle and the intrinsic properties of bone (e.g., elasticity, fracture toughness, viscoelasticity). In this context, the energy balance in a fractured elastic solid (cranial vault) is defined by the relationship between the available energy and the energy consumed in the fracture process. The available energy comes from both external input and the elastic energy stored in the material, while the expended energy is distributed between fracture propagation and dissipation through plastic deformation processes, similarly to depressed fractures where permanent deformation occurs.

The cranial vault exhibits a non-linear mechanical response: under moderate forces it behaves flexibly and absorbs energy efficiently, while at higher forces it becomes more rigid and less capable of dissipating energy^42,56^. This helps explain why absorbed energy does not increase proportionally with peak force. Impact duration further modulates this behaviour, as longer impacts allow greater deformation and energy dissipation. This dependency between energy absorbed and peak force correlations reported by Yoganandan et al. (Fig. 3), highlighting the need to record impact timing because energy absorption is partly governed by deformation rate.

Impactor weight influences fracture mechanics, but its effects depend strongly on the experimental setup. In drop tower systems, weight increases initial kinetic energy but does not influence velocity, which depends solely on drop height. In pneumatic systems, velocity is controlled independently of weight, although weight still contributes to total impact energy. However, its influence appears to be modulated by experimental conditions, as the data in the graphs cluster according to the experimental system used rather than following the trend lines (Figs. 5 and 6). Accordingly, only the relationship between impactor weight and absorbed energy is consistently meaningful, whereas correlations with velocity or peak force are system-dependent and not theoretically causal.

Soft tissues do not appear to substantially influence cranial vault resistance (Fig. 5), although they may contribute to energy distribution and reduction of local stress concentrations as noted by McElhaney, et al^57^.. Bone thickness, by contrast, emerges as a key determinant of peak force resistance. These findings align with those of Cormier, et al^33^., where measurements of bone and overlying soft tissue thickness showed no correlation with fracture force. The fact that soft tissues do not exert significant influence on cranial vault resistance suggests that analyses focused on bone thickness alone may be sufficient to draw valid conclusions about fracture mechanics in archaeological remains. However, anatomical variability in cranial thickness means that local susceptibility to fracture may differ across cranial bones (frontal, temporal or occipital)^32,39,57^. Future research should incorporate additional anatomical variables, such as bone density, to refine our understanding of vault resistance.

Finally, it is worth noting that no fractures were observed in impacts with forces below 2,000 N. These findings align with those of Cormier, et al^33^., who reported a 50% risk of fracture occurring between 1,885 N and 2,405 N for the frontal bone. Similarly, Nahum, et al^49^. and Schneider and Nahum^39^ established a minimum fracture tolerance of 4,003 N for the frontal bone and 2,001 N for the temporoparietal region. It seems logical to assume that higher impact forces increase the probability of fractures ocurring. Therefore, the 2,000 N threshold could be considered a preliminary fracture threshold, provided that the variability between specimens and the specific conditions of the experiments are taken into account.

On the other hand, the analysis of impact surfaces revealed that broad surfaces were primarily associated with the production of linear fractures, as this association could be observed in 88.5% of the analysed cases (*n* = 113). Linear fractures have been widely documented in contexts of accidental falls, sudden death events, and suicides^58–60^. Nevertheless, this type of fracture is also associated with events of interpersonal violence, including assaults involving punches, blows with household objects, or situations where the victim is pushed or impacted against surfaces such as walls or the floor^61,62^. Since linear fractures are common in accidents, suicides, and violent events, their presence alone is insufficient in differentiating between these scenarios. However, these fractures are predominantly characteristic on both direct and indirect impacts with broad surfaces, which explains their occurrence in both types of situations.

Impacts generated by broad surfaces disperse the energy of the blow over a larger area, leading to the formation of more linear fractures while significantly reducing the likelihood of depressed or penetrating ones^63^. This energy dispersion is a key biomechanical factor in explaining the high prevalence of linear fractures in these contexts. It may also account for the greater occurrence of concentric fractures associated with wide-surface impacts, as these fractures, despite being concentric to the primary fracture, are still morphologically linear. Therefore, while linear fractures alone may not be definitive for differentiating trauma mechanisms, they may provide valuable insight into impact surfaces in taphonomic analysis.

Focal impact surfaces were associated with all primary fracture types in similar proportions: notches (38.46%), depressed fractures (30.77%), linear fractures (29.49%), and penetrating fractures (1.28%), out of a total of 78 fractures. Compared to broad impact surfaces, notches and depressed fractures were more frequently observed, whereas linear fractures were less common (Table 1). Notably, the only documented penetrating fracture in the entire dataset was linked to a focal surface. This is due to impacts with focal surfaces concentrating energy in small areas, facilitating the formation of localized injuries, such as depressed or penetrating fractures. In turn, depressed and penetrating fractures are closely associated with events of interpersonal violence and have not been documented in cases of accidental falls or suicides^58,60,64^, so these fracture patterns are characteristic of homicide contexts^65^.

Moreover, impacts with focal surfaces tend to be more violent, concentrated, and high-energy, which not only increases the likelihood of depressed or penetrating fractures but also amplifies the severity and extent of the resulting injuries. This severity is reflected in the high frequency of secondary comminuted fractures associated with focal impacts (Table 2). While comminuted fractures have been documented in accidental falls^60,62^, our analyses indicate that most comminuted fractures are linked to depressed fractures, consistent with previous research^22,26,27,41^. In this sense, our study confirms this association by systematically examining the majority of publicly available experimental data, providing a comprehensive quantitative basis for these patterns. This strong association between depressed fractures, comminuted fractures, and focal impact surfaces reinforces the idea that these fracturing patterns are indicative of interpersonal violence contexts.

From an archaeological perspective, the practical application of this work depends on variables that can be directly observed or inferred from skeletal remains. Bone thickness, fracture morphology, and the presence and distribution of secondary fractures offer indirect but informative proxies for impact energy and surface characteristics, providing a workable framework for interpreting traumatic events. For example, depressed and comminuted fractures with well-defined focal zones suggest high energy impacts involving small contact surfaces, whereas widely dispersed linear fractures are consistent with low energy events or impacts with broad surfaces^22,26,27,41,45^. Importantly, the strong association between depressed and comminuted fractures and focal impacts underscores their value as highly predictive markers of interpersonal violence in the archaeological record. Although the physical parameters of an impact cannot be measured directly in archaeological remains, they can be approximated through controlled experiments using human analogues or synthetic substitutes under comparable loading conditions. Integrating archaeological evidence with experimental data thus enables the reconstruction of relative impact severity and provides a practical framework for applying fracture mechanics criteria to archaeological cases despite the absence of direct biomechanical measurements.

## Conclusion

Impact experiments on human subjects have provided valuable data for understanding the fracture mechanics of the cranial vault. However, an analysis of the existing databases revealed significant discrepancies between studies due to methodological differences, which hinder the integration of results and limit their applicability to the archaeological record. A key factor in this variability is the use of peak force as a measure of impact magnitude, as this variable depends on the cranial vault’s structural resistance and introduces uncontrolled factors that complicate comparisons between studies. In contrast, energy has proven to be a more accurate indicator for assessing impact magnitude, highlighting the importance of its measurement in future experimental studies.

Despite these limitations, the analysed data suggest that fractures do not occur in impacts with forces below 2000 N. This value could be considered a preliminary fracture threshold; however, it should be interpreted with caution, considering variability among samples and experimental conditions. To improve the quality and comparability of future studies, the application of dynamic fracture mechanics principles is recommended to identify the most relevant variables. In this regard, priority should be given to measuring impact force rather than peak force, as it is independent from the structural characteristics of the cranial vault. Additionally, recording impact duration is essential, given that the vault’s energy absorption capacity does not follow a linear relationship.

Moreover, the analysis of bone thickness and soft tissues revealed that the latter do not significantly influence the cranial vault’s biomechanical resistance to impacts, whereas bone thickness has a notable effect. This finding suggests that impact resistance primarily depends on the bone itself, making bone thickness a fundamental variable in both experimental studies and the analysis of archaeological remains. However, soft tissues may play a relevant role in redistributing forces across the cranial surface, highlighting the importance of considering them in future studies on impact biomechanics.

The fracture pattern analysis revealed a strong relationship between broad impact surfaces and linear fractures, providing a key criterion for differentiating impact surfaces in taphonomic analyses. In contrast, focal impact surfaces were notably associated with depressed and comminuted fractures. Although comminuted fractures may be caused by accidental falls, their link to depressed fractures suggests a different origin, often associated with interpersonal violence. The high incidence of cranial fractures in these contexts reinforces the significance of depressed fractures, particularly multiple or comminuted, as a reliable marker of focal impacts and a key indicator of violence in forensic and archaeological studies.

These findings highlight the importance of applying a Fracture Mechanics approach to the study of the archaeological record. In this regard, evaluating the possible impact surfaces responsible for a fracture can provide valuable insights for characterizing fracture patterns, although it may not always be possible in archaeological contexts due to incomplete contextual information. This approach allows for a more comprehensive understanding of the mechanisms involved in fracture formation and contrasts with interpretations based solely on the isolated analysis of fractures, which, in many cases, are insufficient to distinguish between intentional violence and accidental events. To advance this field, studies that more precisely characterize fracture mechanics will be required.

## Materials and methods

### Database built

In order to build the dataset, we selected those studies that conducted blunt dynamic impact experiments on human subjects. This meta-analysis was based solely on data extracted from prior peer-reviewed publications. No direct experimentation or handling of cadaveric material was performed by the authors.

The studies were selected if they provided data on physical variables associated with the impact and if they documented pathological findings, or specified that all subjects had experimented fractures or not^26,27,34–36,39–42,45,49,55,66–71^ (Supplementary text 2). In instances where a subject sustained multiple impact to different regions of the cranial vault, or in cases where multiple impacts were sustained in succession until the vault was fractured, the impacts were considered on an individual basis, in the dataset.

In this study, we focus on the cranial vault bones, which consist of the frontal, parietal, temporal, sphenoid, and occipital bones. Among these, the parietal, occipital, and frontal bones are particularly significant due to their structural composition: they feature outer and inner tables of dense cortical bone separated by a trabecular diploë. This unique architecture makes these bones more diagnostic for interpreting fracture patterns compared to thinner cranial bones, such as those of the face. Consequently, our analysis focuses on fractures occurring in these cranial vault bones.

To ensure the relevance and accuracy of our findings, we excluded those cases that did not employ human heads or cadavers, those focusing on other regions of the cranium (e.g., the mandible, maxilla, or zygomatic arch), experiments using animal subjects, and studies that failed to characterise fractures or relied on padding or quasistatic conditions^56,72–77^. In addition, Nusholtz, et al^78^. were also excluded because the data provided was unclear and difficult to interpret.

### Physical and anatomical variables analysis

The selected physical parameters included were those related to impact events: absorbed energy, peak force, loading velocity, and impactor weight. Drop height was also included in cases where a drop tower was implemented. Consequently, studies that measured other types of variables were excluded, such as the kinetic energy of the cranial vault before impact, not the absorbed energy^45^. Finally, the variables using imperial units were converted to the decimal metric system.

- **Absorbed energy**: Energy retained or dissipated by the cranial vault and surrounding soft tissues when subjected to an impact, lasting until the load ceases or a fracture occurs. This energy represents the portion of the initial impact energy that is not returned to the set-up.
- **Peak force**: Maximum force recorded during a dynamic event. It represents the highest level of applied force that is transmitted through the cranial vault until it is fully dissipated or until a fracture occurs.
- **Loading velocity**: Speed at which a force is applied to the cranial vault over a given time interval upon impact.
- **Impactor weight**: Mass of the object used to generate the impact.
- **Drop height**: Vertical distance from which an object is dropped to the point of impact.

These variables were selected for two reasons: firstly, because they were consistently reported in most of the research, and secondly, because they reflect the ability of the cranial vault to mitigate an impact or fracture. We acknowledge, however, that other factors, such as cranium geometry, bone anisotropy, and suture flexibility, also influence fracture propagation and morphology and should be considered in future studies beyond the variables analyzed here.

An increase in velocity will result in an elevated risk of injury due to the generation of a higher peak force and, consequently, increased kinetic energy, which will impair the capacity of the cranial vault to absorb and dissipate this energy^79^. The weight of the impactor is another critical factor; heavier impactor will produce a greater kinetic energy at impact, contributing to an escalation in both peak force and absorbed energy. However, while increasing the mass results in a linear increase of kinetic energy, increasing the velocity results in a parabolic (quadratic) increase in kinetic energy. Accordingly, while mass is a contributing factor, the potential for injury is primarily determined by the velocity at the time of impact^43,44^.

Many of the articles reviewed did not provide detailed explanations of the measurement or data calculation methods used. Therefore, those articles that provided data on more than one variable were used to analyse the correlation between loading velocity, energy absorbed, peak force and impactor weight^26,27,39–42^. The aim was to ensure the comparability of the data between different experiments and to assess their applicability to the archaeological record. The presence of correlations between these variables across experiments would indicate a potential similarity in the methods used to measure and/or calculate the data.

In the case of Fenton, et al^41^., it was possible to calculate the velocity for the tests using an impact tower because they were dealing with a uniformly gravitationally accelerated rectilinear motion. Similarly, whenever possible, we recalculated the loading velocities reported in other studies^34–36^. The velocity was calculated as:

$$\:v=\sqrt{2\:g\:h}$$

where g refers to the acceleration of gravity, and h, the height the object is from the origin of distances (floor).

Finally, whenever possible, the bone and soft tissue thickness was analysed to determine their contribution to the cranial vault’s impact resistance. Bone thickness is recognized as a critical factor in energy absorption and dissipation during dynamic impacts, as it directly influences the structural integrity of the cranial vault^27^. However, the role of soft tissues, such as the scalp, has been subject to debate in the literature. While some studies suggest that soft tissues significantly mitigate impact forces by acting as a cushioning layer^45–47,49^, others argue that their energy absorption capacity is relatively low and that they may be more closely related to the redistribution of forces rather than direct energy absorption^57,70^. By including these measurements in our analysis, we aim to contribute to this ongoing debate and provide a more nuanced understanding of how both bone and soft tissue thickness influence the biomechanical response of the human cranial vault to blunt impact conditions.

### Fracture analysis

Each impact included in the dataset described one, multiple, or no fractures. In cases where multiple fractures were documented in a single impact event, only the primary fracture associated with the impact variables that resulted in the total number of fractures was considered in the primary fracture analysis (e.g., if an impact resulted in a depressed fracture at the point of impact in addition to associated linear fractures, the depressed fracture was the only one taken into consideration). The purpose of this classification is twofold: first, to facilitate analysis, and second, to initiate discussion of the relationship between certain primary fracture types and certain variables and impact surfaces. For the analysis of fractures and impact surfaces, we used all of the above-mentioned papers, with the exception of Kroman, et al^55^., which did not provide information on the type of impact surface used.

In order to provide a unified classification of fractures, a criterion was developed by integrating the analysed articles with classification standards from forensic and medical sources^3,22,26,27,31,39,46,48–54,80^ (Supplementary Figs. 9–11). Therefore, those original fracture classifications that did not report sufficient data or photographs for their interpretation were excluded, such as severe fractures, compound fractures, elliptical fractures, and massive circular fractures, among others (Supplementary text 2). In these cases, we accompanied the description of the fracture with the following term: “Interpretation is not possible.”

Because it was not possible to determine the exact impact area of each impactor directly, an alternative approach was taken to categorize the impactors into two groups: focal and broad. The total area of each impactor was calculated with the assumption that focal impactors would have smaller impact areas than broad impactors. Where available, photographs of the impactors were also used to assist in this classification (Supplementary text 2).

## Supplementary Information

Below is the link to the electronic supplementary material.

Supplementary Material 1

Supplementary Material 2

## Data Availability

The datasets generated are included in the Supplementary Information.
