# Supplementary material for: Bioengineering approaches to dynamic impact analysis for cranial fracture interpretation in arcaheology
Source: Sci Rep. 2026 Feb 11;16:8327. doi: 10.1038/s41598-026-38313-0 (PMC12966295; doi:10.1038/s41598-026-38313-0)
Supplement: Supplementary file 2 — Supplementary Material 2 [file 41598_2026_38313_MOESM2_ESM.docx]

**BIOENGINEERING APPROACHES TO DYNAMIC IMPACT ANALYSIS FOR CRANIAL FRACTURE INTERPRETATION IN ARCAHEOLOGY**

Daniel Rodríguez-Iglesias^1*^, Ana Pantoja-Pérez^1,2^, Ángel De La Rosa^3^, Pedro Latorre-Carmona^4^, Nohemi Sala^1,2^

1 Centro Nacional de Investigación sobre Evolución Humana (CENIEH), Burgos, Spain.

2 Centro UCM-ISCIII de Evolución y Comportamiento Humanos, Avd/ Monforte de Lemos, 5, Pabellón 14, 28029 Madrid, Spain.

3 Departamento de Tecnología Química, Energética y Mecánica, DIMME, Grupo de Durabilidad e Integridad Mecánica de Materiales Estructurales, Universidad Rey Juan Carlos, Madrid, Spain.

4 Departamento de Ingeniería Informática, Universidad de Burgos, Avda. Cantabria s/n, Burgos, 09006, Spain.

* Corresponding author: [r.iglesiasdaniel@gmail.com](mailto:r.iglesiasdaniel@gmail.com) / <https://orcid.org/0000-0002-9644-7477>

**SUPPLEMENTARY INFORMATION**

**Supplementary Information 1: Summary of experimental human impact tests**

| **Set-up** | **Impactor area (cm2)** | **Peak Force** | | **Loading Velocity** | | **Absorbed Energy** | | **Sample Size** | **Reference ^a^** |
| --- | --- | --- | --- | --- | --- | --- | --- | --- | --- |
|  |  | **Range (N)** | **Mean (N)** | **Range (m/s)** | **Mean (m/s)** | **Range (J)** | **Mean (J)** |  |  |
| Drop tower | 6.45 circular disk | 2215.21 - 8851.96 | 4207.77 | - | - | - | - | 36 | 8 |
| Drop tower | 6.45 circular disk | 2120 - 9880 | 4667.78 | 4.87 - 5.97 | 5.58 | 16.2 - 56.5 | 40.17 | 27 | 7 |
| Drop tower | 72.38 hemisphere anvil | 8809 - 14034 | 11937.67 | 7.1 - 8 | 7.5 | 14.06 - 43.48 | 28.02 | 6 | 4 |
| Drop tower | - | 5475.75 - 6271.99 | 5991.75 | - | - | - | - | 3 | 26 **^b^** |
| Pneumatic piston | 20.27 hemispherical implement | 4655 - 9532.6 | 6849.67 | 6.75 - 7.23 | 6.92 | 95.2 - 134.3 | 113.93 | 3 | 5, 12 |
| Drop tower | 20.27 spherical implement | 5469.2 - 5966.1 | 5717.65 | 2.43 - 2.84 | 2.63 | - | - | 2 | 5 |
| Drop tower | 6.45 square flat implement | 5214 - 7282.8 | 5908.1 | 2.84 - 3.57 | 3.19 | - | - | 3 | 5 |
| Pneumatic piston impactor | 6.45 square flat implement | 12498.8 - 14137.3 | 13425.97 | 6.78 - 7.58 | 7.27 | 122.5 - 139.3 | 129.87 | 3 | 5, 12 |
| Pneumatic piston | 45.6 flat implement | 7087.7 - 8904 | 8198.8 | 6.73 - 8.14 | 7.48 | 117.5 - 152.7 | 135.63 | 4 | 5, 12 |
| Drop tower | 6.45 square flat implement | 5351.1 - 11826.2 | 7611.63 | 2.73 - 3.81 | 3.26 | - | - | 4 | 5 |
| Drop tower | 45.6 flat implement | 8359.9 - 8981.7 | 8703.87 | 3.19 - 4.83 | 3.93 | - | - | 3 | 5 |
| Pneumatic piston | 126.72 cylinder oriented horizontally | 4824.4 - 8581.9 | 6281.54 | 5.77 - 6.33 | 6.09 | 49.5 - 130.27 | 78.38 | 8 | 5, 12 |
| Pneumatic piston | 45.6 broad flat surface | 3695 - 10806.77 | 7311.89 | 4.93 - 6.44 | 5.79 | 43.9 - 115.38 | 74.41 | 8 | 5, 12 |
| Pneumatic piston | 6.45 focal surface | 4178.27 - 10855.6 | 6501.45 | 5.79 - 6.51 | 6.02 | 40.47 - 78.59 | 59.5 | 8 | 5, 12 |
| Drop tower | 15.7 semi-circular rod-shaped implement | 2600 - 8600 | 4715.38 | - | - | - | - | 13 | 22 |
| Drop tower | 5 flat circular plate | 2500 - 10000 | 5195 | - | - | - | - | 11 | 23 |
| Drop tower | 52 rectangular plate | 5800 - 17000 | 12390.91 | - | - | - | - | 20 | 23 |
| Pneumatic piston | 176.71 of wide and flat surface | 4655 - 11662 | 8952 | 4.1 - 6.2 | 5.36 | - | - | 7 | 24 |
| Pneumatic piston | 181.46 implement | 7150 - 9610 | 8783.33 | 6.35 - 7.17 | 6.78 | - | - | 3 | 20 |
| Two-pendulum | 38.48 flat-surfaced cylindrical | - | - | 5.3 - 5.4 | 5.33 | 13.44 - 17.91 | 16.37 | 3^b^ | 25 |
| Two-pendulum | 38.48 flat-surfaced cylindrical | 5938 - 14269 | 10419.31 | 3.39 - 6.99 | 5.6 | 8.98 - 40.48 | 22.87 | 13 | 6 |
| Free fall | A flat, rigid metal plate | 12200 - 12500 | 12350 | - | - | - | - | 2 | 21 |
| Free fall | Steel slab | - | - | 4.11 - 6.95 | 5.35 | - | - | 46 | 17 |

**Table S1.** Summary of experimental human impact tests that resulted in fractures. Key parameters: impactor area, peak force, loading velocity and absorbed energy are shown, together with sample size and corresponding literature references. a Hodgson's studies were excluded because errors in calculations were identified [^1-3^](#_ENREF_1). b Head number 4 was excluded from the table because only velocity data were reported.

| **Set-up** | **Impactor area (cm^2^)** | **Peak Force** | | **Loading Velocity** | | **Energy Absorbed** | | **Sample Size** | **Reference^a^** |
| --- | --- | --- | --- | --- | --- | --- | --- | --- | --- |
|  |  | **Range (N)** | **Mean (N)** | **Range (m/s)** | **Mean (m/s)** | **Range (J)** | **Mean (J)** |  |  |
| Drop tower | 6.45 cm^2^ circular disk | 1699.22 - 7117.15 | 4036.76 | - | - | - | - | 4 | 8 |
| Drop tower | 6.45 cm^2^ circular disk | 1340 - 7740 | 4003.5 | 3.86 - 5.98 | 5.37 | 11.2 - 56 | 36.11 | 21 | 7 |
| Drop tower | - | 6227.51 | - | - | - | - | - | 1 | 26 |
| Pneumatic piston | 176.71 cm2 of wide and flat surface | 4851 - 9138 | 7178.33 | 2.8 - 6.1 | 4.87 | - | - | 3 | 24 |
| Pneumatic piston | 181.46 cm2 implement | 14600 | - | 6 | - | - | - | 1 | 20 |
| Two-pendulum | 38.48 cm2 flat-surfaced cylindrical | - | - | 2 - 3.8 | 3.2 | 10.16 - 35.86 | 19.02 | 3 | 25 |

**Table S2.** Summary of experimental human impact tests that did not result in fractures. Key parameters: impactor area, peak force, loading velocity and absorbed energy are shown, together with sample size and corresponding literature references. a Hodgson's studies were excluded because errors in calculations were identified [^1-3^](#_ENREF_1)

| **Set-up** | **Variables** | **Number** | **Coefficient of Determination** | **p-value** | **Blow Site** | **Reference** |
| --- | --- | --- | --- | --- | --- | --- |
| Drop tower | Absorbed energy – Peak force | 47 | *R² =* 0.21 | *p* < 0.001 | Temporo-parietal and frontal | 7 |
| Two-pendulum |  | 13 | *R² =* 0.39 | *p* = 0.023 | Frontal | 6 |
| Drop tower |  | 6 | *R² =* 0.06 | *p* = 0.635 | Occipital, frontal and vertex | 4 |
| Pneumatic system |  | 34 | *R² =* 0.27 | *p* < 0.001 | Parietal | 5, 12 |
| Comparative analysis |  | 81 | *R² =* 0.47 | *p* < 0.001 | Temporo-parietal, frontal and parietal | 5, 7, 12 |
| Drop tower | Absorbed energy – Loading velocity | 47 | *R² =* 0.27 | *p* < 0.001 | Temporo-parietal and frontal | 7 |
| Pneumatic system |  | 34 | *R² =* 0.69 | *p* < 0.001 | Parietal | 5, 12 |
| Drop tower |  | 6 | *R² =* 0.29 | *p* = 0.274 | Occipital, frontal and vertex | 4 |
| Two-pendulum |  | 13 | *R² =* 0.05 | *p* = 0.451 | Frontal | 6 |
| Comparative analysis |  | 81 | *R² =* 0.71 | *p* < 0.001 | Temporo-parietal, frontal and parietal | 5, 7, 12 |
| Pneumatic system | Peak force – Loading velocity | 38 | *R² =* 0.14 | *p* = 0.026 | Parietal | 5, 12 |
| Drop tower |  | 12 | *R² =* 0.36 | *p* = 0.038 | Parietal | 5 |
| Drop tower |  | 47 | *R² =* 0.05 | *p* < 0.001 | Temporo-parietal and frontal | 7 |
| Two-pendulum |  | 13 | *R² =* 0.04 | *p* = 0.53 | Frontal | 6 |
| Drop tower |  | 6 | *R² =* 0.23 | *p* = 0.34 | Occipital, frontal and vertex | 4 |
| Comparative analysis |  | 81 | *R² =* 0.3 | *p* < 0.001 | Temporo-parietal, frontal and parietal | 5, 7, 12 |
| Drop tower | Absorbed energy – Impactor weight | 47 | *R² =* 0.83 | *p* < 0.001 | Temporo-parietal and frontal | 7 |
| Pneumatic system |  | 27 | *R² =* 0.52 | *p* < 0.001 | Parietal | 5, 12 |
| Comparative analysis |  | 73 | *R² =* 0.72 | *p* < 0.001 | Temporo-parietal, frontal and parietal | 5, 7, 12 |
| Drop tower | Peak force – Impactor weight | 47 | *R² =* 0.23 | *p* < 0.001 | Temporo-parietal and frontal | 7 |
| Pneumatic system |  | 27 | *R² =* 0.02 | *p* = 0.504 | Parietal | 5, 12 |
| Drop tower |  | 12 | *R² =* 0.12 | *p* = 0.261 | Parietal | 5 |
| Comparative analysis |  | 73 | *R² =* 0.31 | *p* < 0.001 | Temporo-parietal, frontal and parietal | 5, 7, 12 |
| Pneumatic system | Loading velocity – Impactor weight | 27 | *R² =* 0.34 | *p* < 0.001 | Parietal | 5, 12 |
| Drop tower |  | 12 | *R² =* 0.37 | *p* = 0.0356 | Parietal | 5 |
| Drop tower |  | 47 | *R² =* 0.03 | *p* = 0.268 | Temporo-parietal and frontal | 7 |
| Comparative analysis |  | 73 | *R² =* 0.37 | *p* < 0.001 | Temporo-parietal, frontal and parietal | 5, 7, 12 |
| - | Peak force – Bone thickness | 33 | *R² =* 0.3 | *p* < 0.001 | Frontal, occipital and parietal | 1, 6, 24 |
| - | Absorbed energy – Soft tissues | 14 | *R² =* 0.0001 | *p* = 0.96 | Frontal | 6, 25 |
| - | Peak force – Soft tissues | 23 | *R² =* 0.042 | *p* = 0.347 | Frontal | 6, 24 |

**Table S3.** Comparative summary of all correlation analyses included in this study. The table reports, for each study: set-up, variables tested, sample size, coefficient of determination, p-value and blow site.

**Supplementary Information 2: Physical and anatomical variables correlations**

**
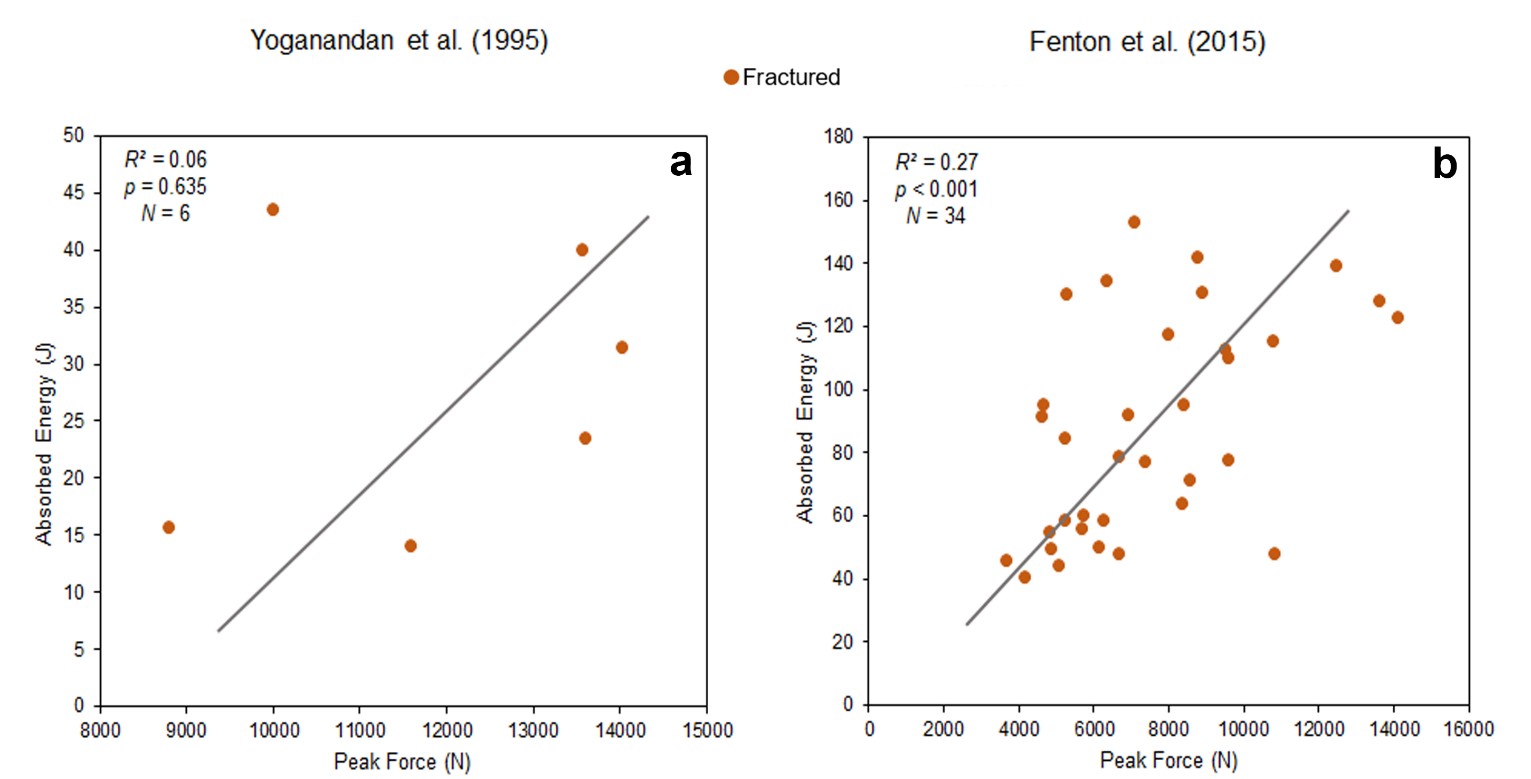
**ABSORBED ENERGY *vs* PEAK FORCE

**Figure S1.** Correlation plots between absorbed energy and peak force. a) Study carried out by [Yoganandan, et al. ^4^](#_ENREF_4) using a drop tower set up. b) Study carried out by [Fenton, et al. ^5^](#_ENREF_5) using a pneumatic system set-up. The plot was generated using the data provided in the paper.

**
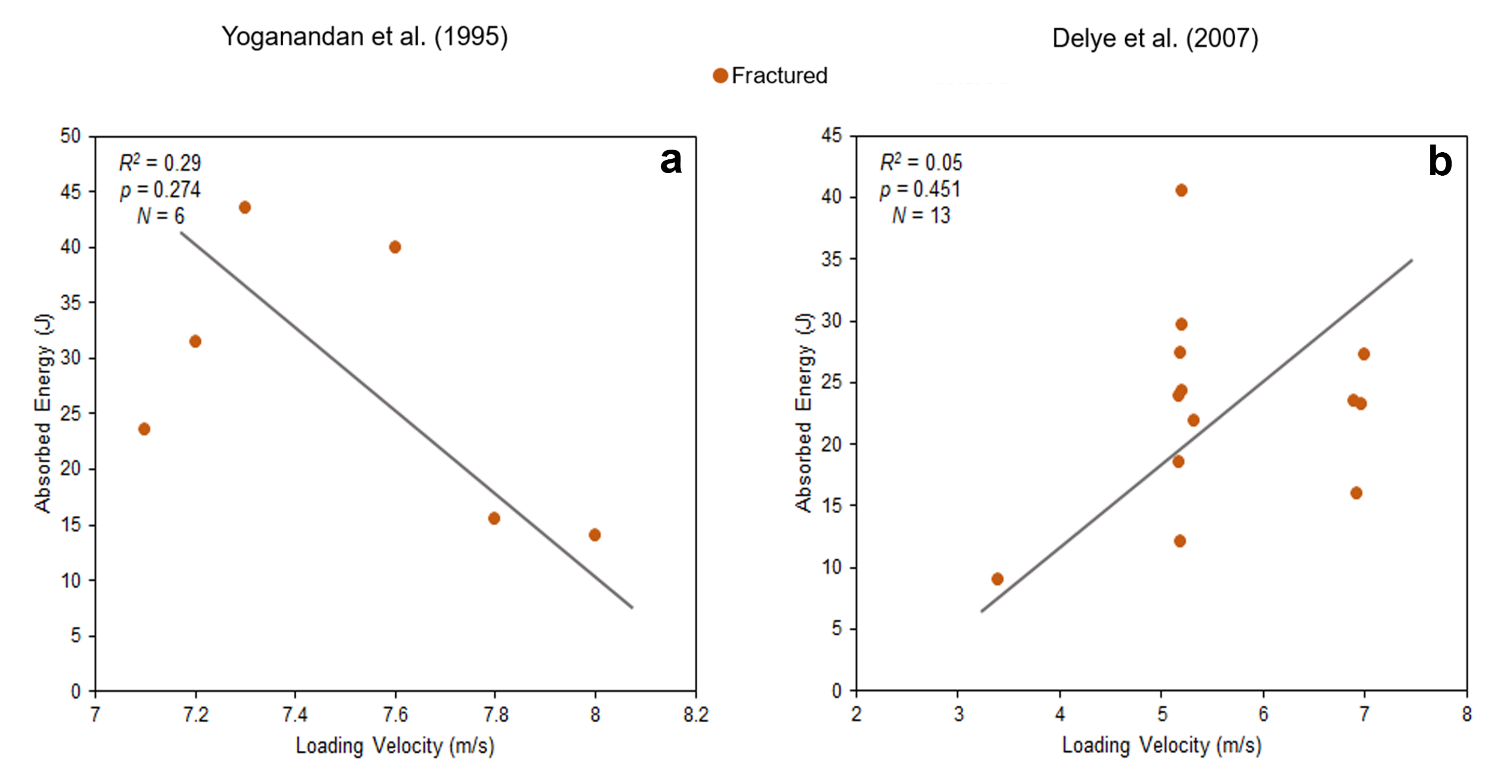
**ABSORBED ENERGY *vs* LOADING VELOCITY

**Figure S2.** Correlation plots between absorbed energy and loading velocity. a) Study carried out by [Yoganandan, et al. ^4^](#_ENREF_4) using a drop tower set up. b) Study carried out by [Delye, et al. ^6^](#_ENREF_6) using pendulum set-up. The plots were generated using the data provided in the papers.

PEAK FORCE vs LOADING VELOCITY

**
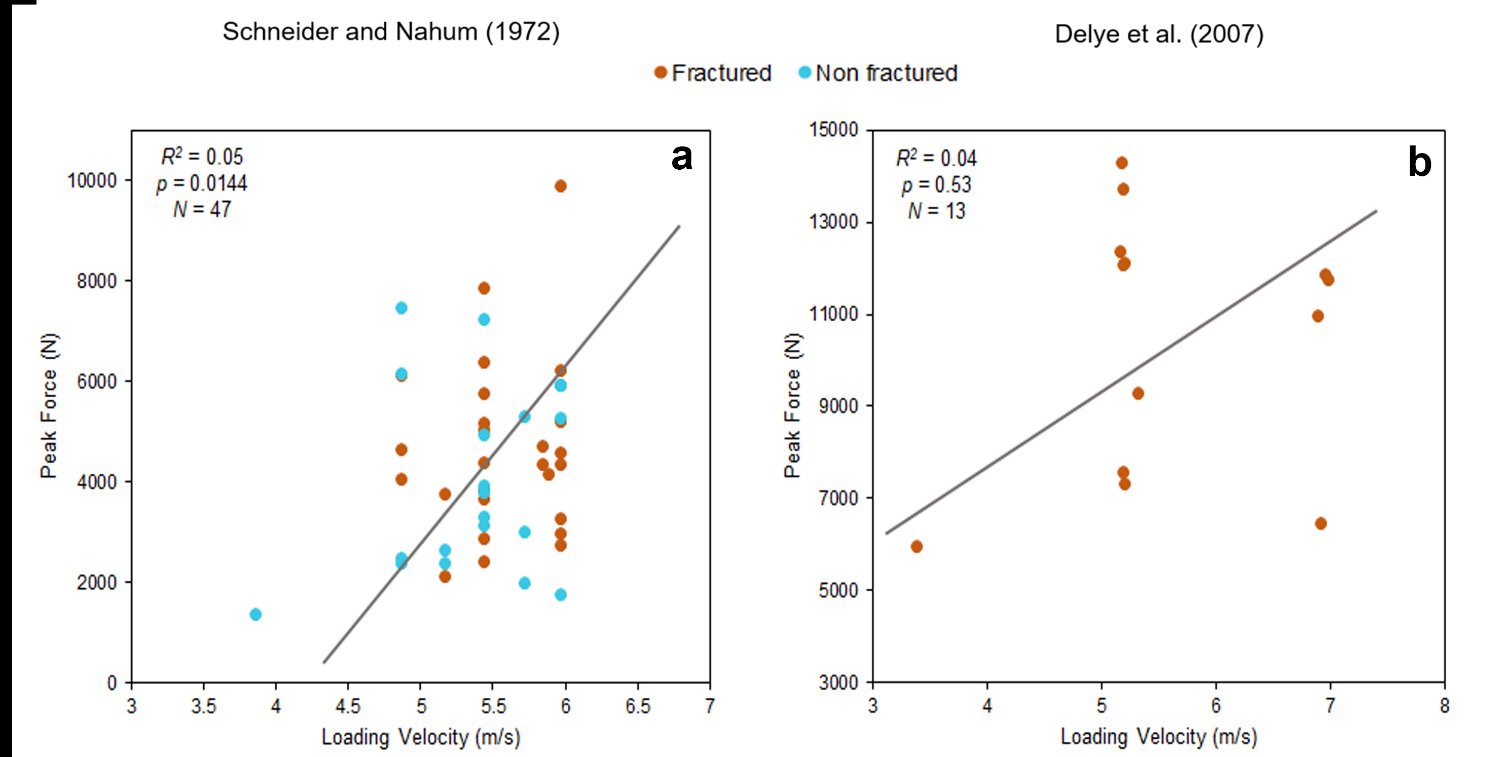


Figure S3.** Correlation plots between absorbed energy and loading velocity. a) Study carried out by [Schneider and Nahum ^7^](#_ENREF_7) using a drop tower set up. b) Study carried out by [Delye, et al. ^6^](#_ENREF_6) using pendulum set-up. The plots were generated using the data provided in the papers.


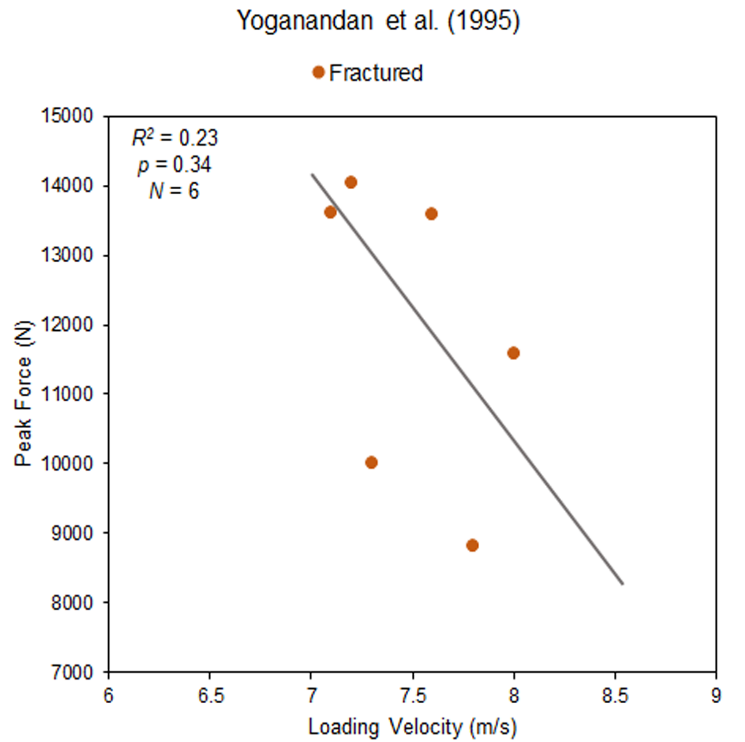
 **Figure S4.** Correlation plot between peak force and loading velocity. a) Study carried out by [Yoganandan, et al. ^4^](#_ENREF_4) using a drop tower set-up. The plots were generated using the data provided in the papers.


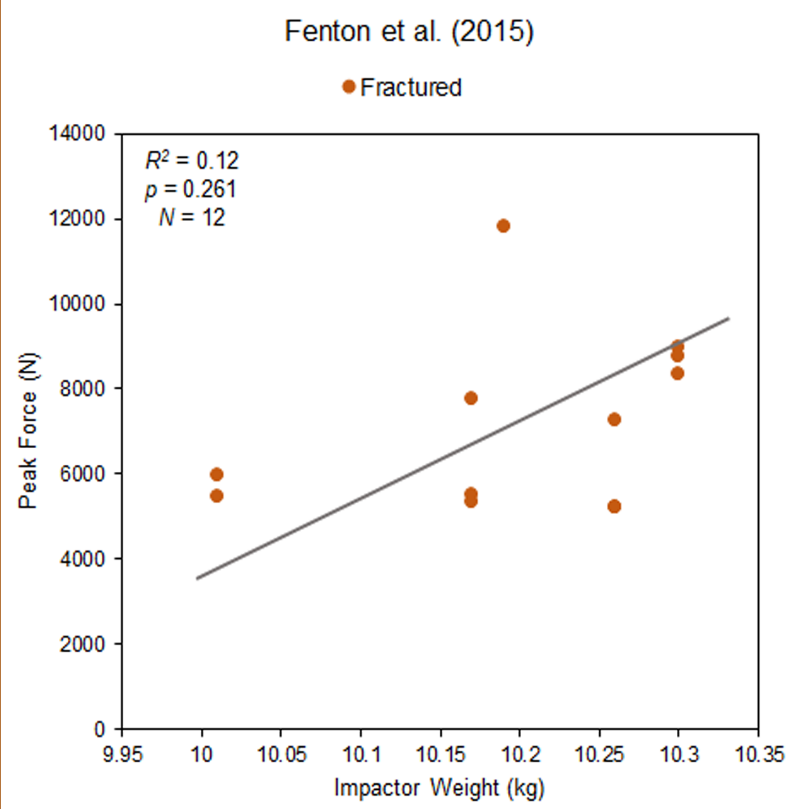
PEAK FORCE *vs* IMPACTOR WEIGHT

**Figure S5.** Correlation plot between peak force and impactor weight. Study carried out by [Fenton, et al. ^5^](#_ENREF_5) using a drop tower set-up. The plot was generated using the data provided in the paper.

**
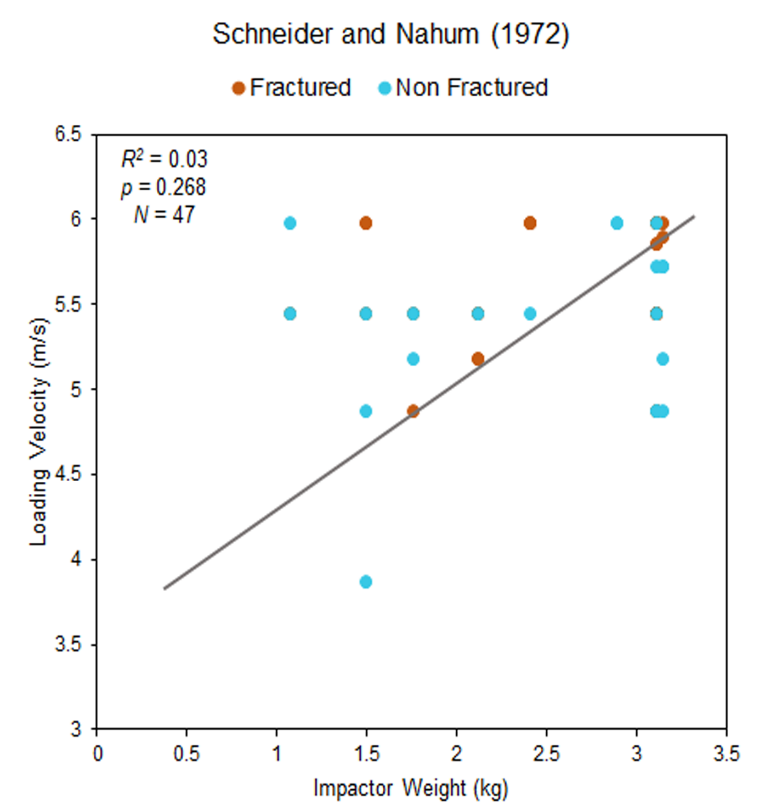
**IMPACTOR WEIGHT *vs* LOADING VELOCITY

**Figure S6.** Correlation plot between loading velocity and impactor weight. Study carried out by [Schneider and Nahum ^7^](#_ENREF_7) using a drop tower set-up. The plot was generated using the data provided in the paper.

**
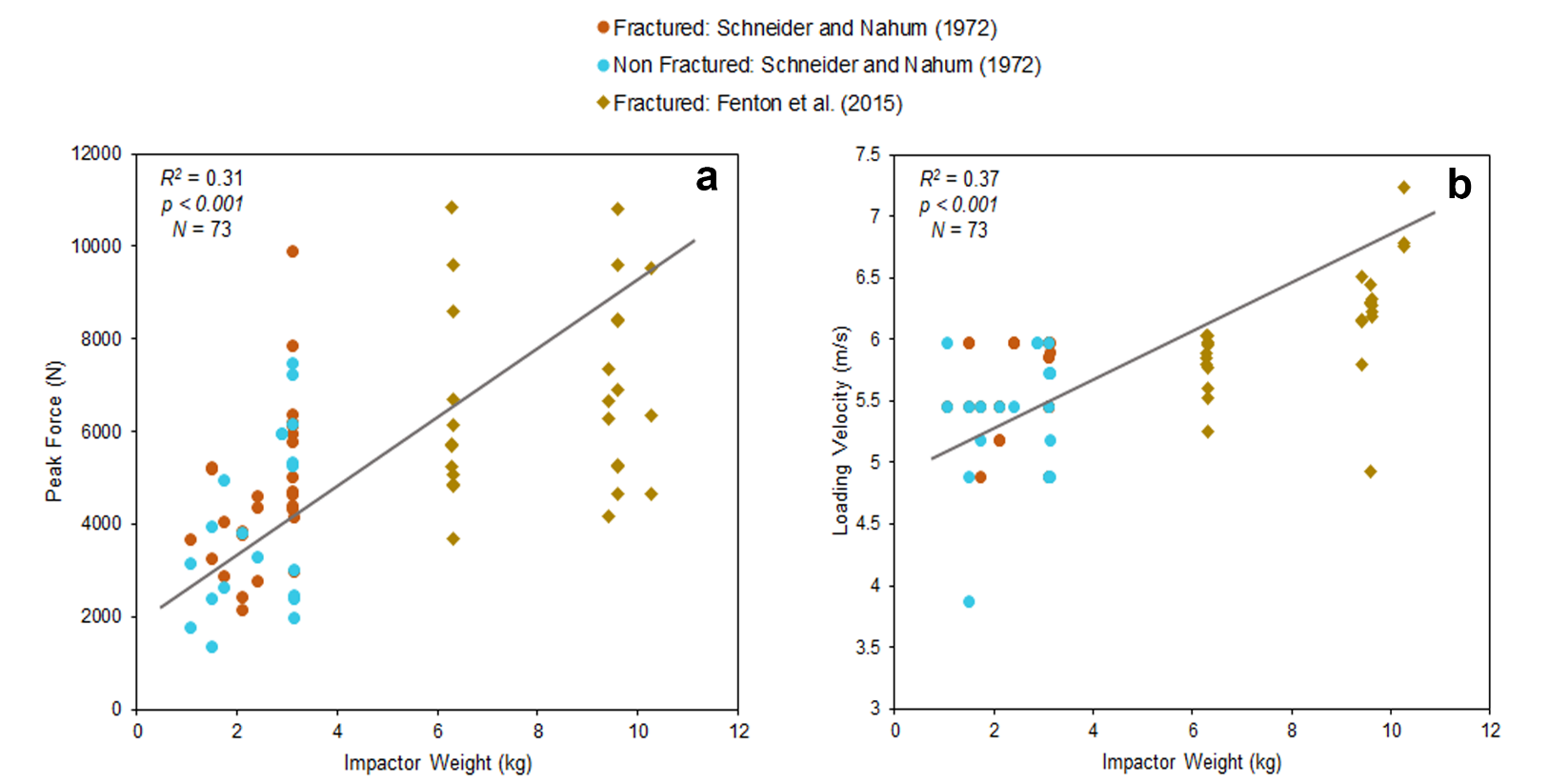
**COMPARATIVE ANALYSIS OF PHYSICAL VARIABLES ACROSS STUDIES

**Figure S7.** Comparative analysis of data from [Schneider and Nahum ^7^](#_ENREF_7) using a drop tower set-up and [Fenton, et al. ^5^](#_ENREF_5), published by [Isa, et al. ^20^](#_ENREF_20)^,^[^21^](#_ENREF_21), using a pneumatic system. a) Correlation plot between peak force and impactor weight. b) Correlation plot between loading velocity and impactor weight. The plots were generated using the data provided in the papers.


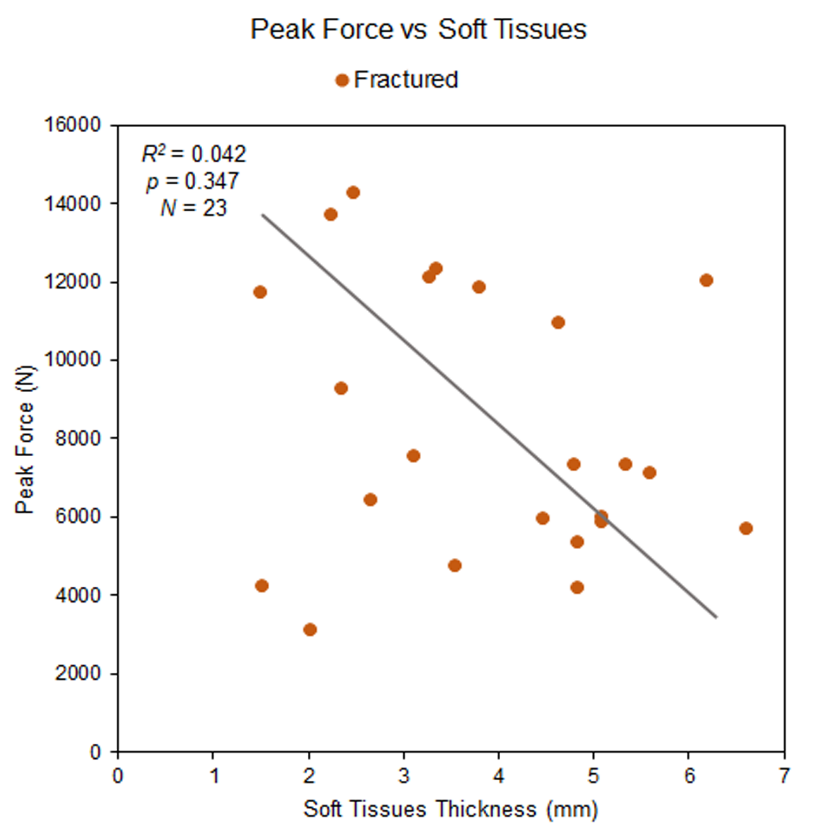

PEAK FORCE VS SOFT TISSUES THICKNESS

**Figure S8.** Correlation plot between peak force and soft tissues thickness.

**Supplementary Information 3**

**Supplementary text 1: fracture criteria**

PRYMARY FRACTURES

Notch fractures are caused by an impact that minimally exceeds the structural strength of the cranial vault. This fracture implies a minimal detectable change, usually a hairline crack or notch, and it is not clinically significant. It represents the minimal energy that is necessary to produce a cranial vault fracture [^7^](#_ENREF_7)^,^[^8^](#_ENREF_8).

Linear fractures are the most common type of cranial fractures and they are typically associated with light to moderate energy impacts distributed over a wide area of the cranial vault [^9^](#_ENREF_9). These fractures involve a single break that passes through the outer and/or inner tables of the bone without a significant displacement. While linear fractures are often considered straight, they rarely follow a simple linear path. The fracture line tends to follow the path of least resistance, with dense bone redirecting the fracture, leading to curvilinear patterns [^10^](#_ENREF_10).

Linear fractures may exhibit a range of morphologies:

- Straight or curvilinear fractures: These include fractures that are linear, semicircular, circular (without depression), or arc-shaped.
- Fissure fractures: In some cases, the separation of the tables and diploë can be significant, resulting in distinct fragments of bone, a condition sometimes referred to as a fissure fracture.
- Diastatic fractures: Case when the fracture extends between two cranial sutures.

These cranial vault fractures may also originate at the impact site, remotely, or both, consistent with the ideas proposed by Gurdjian and colleagues that fractures originate from areas of inbending at the impact site and/or from areas of outbending peripheral to the impact site [^11^](#_ENREF_11)^,^[^12^](#_ENREF_12). Therefore, remote fractures may not extend back to the impact site and may propagate and intersect in adjacent bones. This pattern is typical in temporoparietal blunt force impacts. That is to say, blows to one portion of the cranial vault may cause linear fractures in other areas of the cranium [^12^](#_ENREF_12).

A moderate or high energy impact over a small area will most likely created a depressed fracture [^9^](#_ENREF_9). Depressed fractures are typically caused by impacts concentrated on a small area of the cranial vault, causing the bone of the vault to be displaced endocranially. This may lead to the collapse of the outer table, followed by failure of the diploë and inner table. However, in some cases, the damage may be limited to the outer table and diploë only [^10^](#_ENREF_10)^,^[^13^](#_ENREF_13).

Depressed fractures cause inbending of the cranial vault at the point of impact and peripheral outbending. The outbending can produce radiating fractures that start at one or more points that are distant from the impact site, progressing both toward and away from the impact point. Concentric fractures, which are perpendicular to the radiating fractures, may form as well. This combination of depressed fractures with radial and/or not concentric fractures is sometimes referred to as a stellate fracture [^10^](#_ENREF_10)^,^[^14^](#_ENREF_14). The limiting impact area that determines whether a high-energy impact will result in a depressed or penetrating fracture is 13 cm^2^. If it is larger, the entire cranial vault is stressed, deforms and explodes, resulting in polyfragmentary fracture [^9^](#_ENREF_9).

A penetrating fracture occurs when the force of an object impact is significant enough to penetrate the vault, breaking both the outer and inner tables. This type of fracture can be produced by high-velocity projectiles (e.g. bullets or arrows) or low-velocity projectiles (e.g. knife blades). Both will cause direct damage as they enter the vault; however, some high-velocity projectiles are also able to impart significant kinetic energy to bone fragments and trigger a cavitary shockwave in their wake which can damage tissue at considerable distances from the injury tract. If the object penetrating the cranial vault has sufficient energy, it will be able to create an entry and exit wound. This is known as a perforating fracture. This type of trauma is usually only caused by high-velocity projectiles [^9^](#_ENREF_9)^,^[^15^](#_ENREF_15).

NON-PRIMARY FRACTURES

With a significant amount of force, secondary fractures can appear at close proximity to the impact site. These fractures, known as radial fractures, manifest when the bone is compelled to dissipate additional stress resulting from the impact. Radial fractures are wedge-shaped segments that can follow suture lines or bifurcate surrounding areas of lower cranial strength. The most heavily reinforced areas of the cranial vault may not exhibit radial fractures, and furthermore, an opening of the vault sutures may occur due to the pressure and stress caused by the impact.

Concentric or tertiary fractures are those that occur in the area surrounding the main fracture. This type of fracture can occur close to the main fracture and it appears to be a linear fracture or is associated with radial fractures. Radial fractures tend to relieve the stress on the bone caused by an impact, but they often cause concentric fractures due to the outward displacement of wedge-shaped segments. These fractures are called concentric fractures because they end in the radial cracks, implying that the latter were already present (they are secondary). Since concentric and radial fractures surround the impact point, the wedge-shaped fragments tend to be oriented towards this point, making it easier to identify the impact site [^11^](#_ENREF_11)^,^[^16^](#_ENREF_16).

Lastly, comminuted fractures can occur when a bone breaks into more than two pieces due to low-velocity or high-impact forces, resulting in bone fragmentation. This type of fracture is typically associated with depressed fractures, for instance when the central area is extensively fragmented [^10^](#_ENREF_10). In some cases, this fragmentation may result from the formation of multiple converging concentric, radial, or linear fractures, leading to the fragmentation of the cranial vault. In other instances, fragmentation occurs within the internal region of a depressed fracture or a circularly arranged linear fracture.

**Supplementary text 2: sample information, impactor areas and fracture criteria.**

**GURDJIAN, ET AL.** [**^17^**](#_ENREF_17)

SAMPLE

In the 1940s, [Gurdjian, et al. ^17^](#_ENREF_17), conducted some impact tests to study the mechanics of head injuries. For this purpose, they obtained 55 completely intact human embalmed cadaver heads [^18^](#_ENREF_18). However, they only reported data for 46 specimens.

In these tests, human heads were dropped from heights of 86.36 to 243.84 cm. Impacts were delivered to four positions: 10 specimens were impacted in mid-frontal, 17 specimens in anterior interparietal, 9 specimens in mid-occipital, and 10 specimens in either the right or left posterior parietal.

Each head was weighed and the height from which it was dropped onto the steel slab was measured so the head’s kinetic energy was easily determined using the principle of conservation of mechanical energy. The velocity should have been calculated by the known distance of the fall and the time it took each head to land. Therefore, since this study only reported data for kinetic energy and not absorbed energy, it was excluded from the physical variable’s analysis.

IMPACTOR AREA

Human heads were dropped onto a 72.57 kg steel plate. It is important to note that this surface is a wide impact surface and not a focal surface.

FRACTURE CLASSIFICATION

The fractures were classified as follows:

- Single linear fractures
- Two linear fractures
- Stellate fractures

This classification was adapted according to our specific criteria. Single linear fractures and two linear fractures were grouped into the linear fracture category. Stellate fractures were not related to any of our categories because although this type of fracture implies the appearance of fractures radiating from the point of impact (POI), it can also be related to the appearance of a depressed and concentric fractures [^10^](#_ENREF_10)^,^[^19^](#_ENREF_19).

**NAHUM, ET AL.** [**^8^**](#_ENREF_8)

SAMPLE

In 1968, [Nahum, et al. ^8^](#_ENREF_8) conducted an experiment on human bodies to determine the force required to fracture a human skull in specific regions: frontal, temporo-parietal, and zygomatic.

They used 10 human cadavers for this study. The sample consisted of four males and six females, with an age ranging from 55 to 81 years. Of these cadavers, five were embalmed and five were not. Each cadaver received six blows: two to the frontal region, two to the temporo-parietal region, and two to the zygomatic arch. The impact experiments that were performed on the zygomatic arch were not included in the database.

In order to perform these experiments, they used a drop tower, which allowed for the modification of the impactor’s height or weight. However, height and drop weight are only available for two tests (individual 3 - test numbers 21 and 24). This information appeared in the paper text as an example.

IMPACTOR AREA

The impactor area was described in the paper, using a circular disk of 6.45 cm^2^.

FRACTURE CLASSIFICATION

The fractures produced were divided into the following categories

- 0 = none.
- 1+ = minimal detectable change, usually a hairline crack, not clinically significant.
- 2+ = readily detectable fracture which is clinically significant.
- 3+ = comminuted and/or depressed fractures which represents the extreme of clinical significance.

This classification was adapted to ours, where:

- Notch correspond to: 1+ = minimal detectable change, usually a hairline crack, not clinically significant.
- Linear fracture corresponds to: 2+ = readily detectable fracture which is clinically significant.
- Depressed fracture corresponds to: 3+ = comminuted and/or depressed fractures which represents the extreme of clinical significance

**SCHNEIDER AND NAHUM** [**^7^**](#_ENREF_7)

SAMPLE

In 1972, [Schneider and Nahum ^7^](#_ENREF_7) expanded the cadaver sample to provide new information to extend the tolerance base reported in the preliminary study. In this study, they added new anatomical regions to those already studied: maxilla and mandible.

They used 17 specimens and performed 106 tests. Of these 17 specimens, eight were embalmed and nine were unembalmed, with 11 males and six females ranging in age from 45 to 80 years. Fifteen specimens were impacted twice at the temporo-parietal region, except for specimen 40, which was impacted only once, resulting in 29 tests. Eleven specimens were impacted twice at the frontal region, except for specimens 20, 35, 38, and 42, which were impacted only once, resulting in 18 tests. The remaining 59 tests were conducted on the zygomatic, maxillary, and mandibular regions. These tests were not included in the database.

IMPACTOR AREA

The impactor area was described in the article, using a circular disk of 6.45 cm^2^.

FRACTURE CLASSIFICATION

The criteria for characterizing the fractures produced were the same as those used in [Nahum, et al. ^8^](#_ENREF_8).

**HODGSON, ET AL.** [**^1^**](#_ENREF_1)

SAMPLE

In 1970, [Hodgson, et al. ^1^](#_ENREF_1) conducted an experiment to determine the fracture behaviour of the human frontal bone when hit by two different rigid cylindrical surfaces. According to Table 1, 3, and 4 in the paper, they used 14 cadavers for impact tests, although the abstract mentions that only 12 cadavers were used and they provide information on only 13 tests. In addition, Table 2 mentions that tests 2 and 11 were performed on embalmed heads. Furthermore, the numbering assigned to each cadaver and test (e.g., No. 1 - cadaver 1486) varies across the tables. This variation must be taken into account in order to avoid confusion between the data from different tests. In this case, Table 2 will be used as a reference because it contains the physical data of the impacts performed.

Test number 13 – Cadaver 1492 was excluded because the impact conditions differ; in this case, the body hits the impactor rather than the other way around as in the rest of the tests. With test number 13 excluded, and considering that data for one individual is missing, the age of the 12 individuals included in the database ranged from 33 to 83 years, with 11 males and 1 female.

IMPACTOR AREA

The authors used two types of impactors, both cylindrical in shape, with lengths of 16.51 cm and radiuses of 2.54 cm and 0.79 cm. The impacts were conducted using the lateral surfaces of the cylinders. The side areas were calculated as approximately:

$$A=2*\pi*h=2*\left( \pi\right)*\left( 2.54 \right)*\left( 16.51 \right)= {263.48 cm}^{2}$$

$$A=2*\pi*h=2*\left( \pi\right)*\left( 0.79 \right)*\left( 16.51 \right)=81.95 {cm}^{2}$$

FRACTURE CLASSIFICATION

The fractures produced in these tests were described as either linear or elliptical. According to the authors, the smaller impactor radius (0.79 cm) generated two linear fractures and three localized elliptical fractures, while the larger impactor radius (2.54 cm) produced only linear fractures. The "linear" category corresponds to our definition of linear fractures. However, the term "elliptical" is ambiguous, as it could refer to a linear fracture with a curved morphology or even a depressed fracture with a similar shape. Due to this ambiguity, we chose not to include an interpretation of this fracture type in our analysis.

**HODGSON AND THOMAS** [**^2^**](#_ENREF_2)

SAMPLE

In 1971, [Hodgson and Thomas ^2^](#_ENREF_2) conducted a study with 40 intact, moist, embalmed human cadavers to provide data on human tolerance to skull impact. However, they only provided biological data for 39 individuals, as there is no data for individual number 12. Moreover, in our analysis, 10 tests were excluded for various reasons. Therefore, the sample resulted in 29 individuals: 24 males and five females, ranging in age from 41 to 91 years. The following tests were discarded:

- Test 1 – Cadaver 1717: The accelerometer was loose and the head rotated during impact.
- Test 2 – Cadaver 1745: Problems with measuring instruments.
- Test 5 – Cadaver 1638: Cadaver dehydrated. Test performed to check out equipment.
- Test 15 – Cadaver 1848: The cadaver was dropped from the same height several times.
- Test 17 – Cadaver 1841: The cadaver was dropped from the same height several times.
- Test 18 – Cadaver 1862: The cadaver was used in two experiments.
- Test 19 – Cadaver 1876: The cadaver was dropped from the same height several times.
- Test 27 – Cadaver 1849: The cadaver was dropped from the same height several times.
- Test 28 – Cadaver 154: The cadaver was dropped from the same height several times.
- Test 29 – Cadaver 1873: The cadaver was dropped from the same height several times.
- Test 40 – Cadaver 1932: The cadaver was dropped from the same height several times.

Finally, it should be considered that the data presented in the 1971 report were also included in the 1973 report. However, there are discrepancies regarding the measures of force between the two publications. This may be due to the fact that the 1971 data were updated to include the head injury criteria for both anterior-posterior acceleration (a-p pickup away from the impact site) and head CG resultant acceleration whenever possible, and these data may have been recalculated. For this reason, the physical data yielded by the impacts were collected from the 1973 study, while the biometric data and fracture characterization were collected from the 1971 study, as these variables are not reported in the 1973 study.

IMPACTOR AREA

The authors used four types of impactors: a flat plate, classified as a broad impact surface; a cylinder with a radius of 2.54 cm, assumed to have the same length as the one used in 1970 (16.51 cm), resulting in an area of 263.48 cm²; and two hemispheres with radiuses of 7.62 cm and 20.32 cm, respectively. The areas of the hemispheres were calculated based on their circular bases. All these surfaces were categorized as broad impactors, as supported by the photographs provided. The approximate areas are as follows:

$$A=\pi*r^{2}= \pi{*(7.62)}^{2}=182.41 {cm}^{2}$$

$$A=\pi*r^{2}= \pi*\left( 20.32 \right)^{2}=1,295.13 {cm}^{2}$$

FRACTURE CLASSIFICATION

In order to characterise the generated fractures, authors only describe the lesions using different concepts: localized fracture, linear fracture, massive depressed fracture, massive circular fracture, severe circular fracture, massive compound fracture.

Among these concepts, no interpretations were made for localized fracture, massive circular fracture, severe circular fracture, and massive compound fracture due to insufficient information linking them to our fracture criteria. The term "localized fracture" only indicates the fracture's location. "Compound" and "circular" fractures could potentially refer to either linear fractures or depressed/penetrating fractures.

**HODGSON AND THOMAS** [**^3^**](#_ENREF_3)

SAMPLE

In 1973, [Hodgson and Thomas ^3^](#_ENREF_3) conducted another investigation with 40 embalmed cadavers to fill the gaps in the existing data with respect to head impacts against rigid surfaces, and to provide information about head impacts against deformable surfaces. Thirteen of the cadavers were decapitated, but they do not indicate which ones. All specimens used were male. Twenty-one tests had to be discarded because the cadavers were dropped several times from the same height, the impacts were padded, or no data was reported. This resulted in a total sample of 19 cadavers ranging in age from 47 to 85 years. The following tests were discarded: Test 3 – Cadaver 2108, Test 6 – Cadaver 2095, Test 17 – Cadaver 2258, Test 18 – Cadaver 2220, Test 19 – Cadaver 2214, Test 20 – Cadaver 2205, Test 21 – Cadaver 2247, Test 22 – Cadaver 2219, Test 23 – Cadaver 2439, Test 24 – Cadaver 2358, Test 25 – Cadaver 2365, Test 26 – Cadaver 2440, Test 27 – Cadaver 2353, Test 28 – Cadaver 2483, Test 29 – Cadaver 2354, Test 30 – Cadaver 2412, Test 31 – Cadaver 2425, Test 32 – Cadaver 2448, Test 33 – Cadaver 2212, Test 39 – Cadaver 2068 and Test 40 – Cadaver 2256.

IMPACTOR AREA

The authors utilized five types of impactors. Two cylinders, with radiuses of 2.54 cm and 0.79 cm, were assumed to have the same length as those used in 1970 (16.51 cm), resulting in calculated lateral areas of 263.48 cm² and 81.95 cm², respectively. Two additional cylinders, with radiuses of 1.27 cm and 0.32 cm, were used; however, since no lengths or photographs were provided, it was impossible to calculate their lateral areas or categorize them into broad surfaces, and they were therefore excluded from the analysis. Finally, a rigid hemisphere with a radius of 12.7 cm was also used. The area of the hemisphere was calculated based on its circular base. The approximate area is as follows:

$$A=\pi*r^{2}= \pi{*(12.7)}^{2}=506.71 {cm}^{2}$$

FRACTURE CLASSIFICATION

In most cases, the authors did not characterize the injuries generated after the impacts, instead simply classifying the tests as those that produced fractures and those that did not. However, for test 36 (individual 2326), test 37 (individual 2312), and test 38 (individual 2310), they used the concept of "threshold linear fracture." This characterization criterion was equated to our linear fracture criterion and was incorporated into the data analysis.

**STALNAKER, ET AL.** [**^20^**](#_ENREF_20)

SAMPLE

In 1977, [Stalnaker, et al. ^20^](#_ENREF_20) conducted a study with 15 unembalmed cadavers. Eleven of the impacts were performed with 2.54 cm of Ensolite padding and were thus excluded from the database. Therefore, only four tests were selected (76A144, 76A145, 76A152, and 76A169). Three of these individuals were female, and one was male, ranging in age from 45 to 75 years. The purpose of the tests was to investigate the application of three-dimensional motion analysis using accelerometers, cerebrovascular pressurization, and high-speed cineradiography to better understand the mechanics of head injuries. The following tests were discarded: 75A113, 75A116, 76A126, 76A133, 76A134, 76A135, 76A136, 76A137, 76A159, 76A167 and 76A171.

IMPACTOR AREA

An impactor with a diameter of 15.2 cm was used. The area was calculated as a circle. The area is approximately:

$$A=\pi*r^{2}= \pi{*\left( \frac{15.2}{2} \right)}^{2}={181.46 cm}^{2}$$

FRACTURE CLASSIFICATION

Injuries were evaluated using the AIS revised version in 1976. According to the 1976 AIS, the authors describe two types of fractures for three different individuals.

- Comminuted fracture for individual 17A145.

- Compound and simple fracture for individual 17A152.

- Simple fracture in individual 17A169.

Compound and simple fractures refer to the presence of a fracture with or without an overlying scalp laceration, which may be linear or depressed. Our classification criteria do not consider the presence of soft tissue (which is a criterion applicable to the archaeological record). Therefore, these criteria cannot be applied to our classification. However, they were included in the database because the impact produced a cranial fracture.

On the other hand, comminuted fractures never occur in an isolated manner. They are always associated with a depressed fracture. In this case, no further information is provided. Therefore, this test was included in the database only as a comminuted fracture.

**GOT, ET AL.** [**^21^**](#_ENREF_21)

SAMPLE

In 1978, [Got, et al. ^21^](#_ENREF_21) reported the results of 42 tests involving direct head impacts on fresh, unembalmed, perfused cadavers. These tests included variations with and without helmets, as well as with and without padding, utilizing a free-fall procedure. The objective was to develop a method for investigating the resistance of the human brain to impacts using unembalmed, perfused cadavers. Three main types of impacts were investigated: frontal, temporal-parietal, and frontal-facial. From these experiments, we selected the impacts performed without helmets and without padding. The tests included were: Nº68, Nº76. The tests discarded were: 63, 64, 65, 66, 67, 69, 70, 73, 74, 83, 85, 86, 87, 88, 89, 90, 91, 92, 94, 95, 99, 100 ,101, 102, 103, 104, 107, 108, 109, 110, 111, 134, 140, 141, 142, 143, 144, 145, 146 and 147.

IMPACTOR AREA

The surface used was a flat rigid metal plate. The authors did not provide data on size, but since it was a free fall test it can be categorized into the large impactor group.

FRACTURE CLASSIFICATION

The description of lesions follows the Abbreviated Injury Scale, although the specific version used is not mentioned. Since the report is part of the proceedings of the 22nd Stapp Car Crash Conference, held October 24-26, 1978, it is assumed that the authors used the updated 1976 version of the Abbreviated Injury Scale.

Among the selected cases, two types of fractures were described: minor fractures and very large fractures. These fractures could not be interpreted due to insufficient information, making it unclear whether they represent linear fractures or another type.

**ALLSOP, ET AL.** [**^22^**](#_ENREF_22)

SAMPLE

In 1988, [Allsop, et al. ^22^](#_ENREF_22) conducted a study with the aim of identifying the large-deformation response of the human skull and augmenting the data for small diameter cylindrical facial impacts. For this purpose, they used 15 unembalmed cadaver heads, comprising 11 females and four males, with ages ranging between 39 and 90 years.

Two impacts per head were conducted to obtain the maximum amount of data per specimen: one to the midface region, targeting either the maxilla or zygoma, followed by an impact to the frontal region. We only included data from frontal impact experiments in the database; therefore, impact tests performed on the zygomatic arch were excluded.

IMPACTOR AREA

The impactor used was semi-circular and rod-shaped, 20 mm in diameter and 230 mm in length. The area corresponds to a semi-circular cross-section. The area is approximately:

$$A= \frac{\pi* r^{2}}{2}=\frac{\pi*{10}^{2}}{2}=\frac{3.1416* 100}{2}=15.08 {cm}^{2}$$

FRACTURE CLASSIFICATION

The authors did not use specific criteria to characterise the fractures they observed. However, they reported applying sufficient energy to produce significant fractures in the specimens. Hairline fractures were interpreted using our notch criterion, as they represent minimal, detectable changes, typically in the form of minor cracks with limited clinical relevance. Conversely, severe fractures could not be categorized according to our criteria due to a lack of detailed information provided by the study.

**ALLSOP, ET AL.** [**^23^**](#_ENREF_23)

SAMPLE

In 1991, [Allsop, et al. ^23^](#_ENREF_23) conducted impact tests on 31 unembalmed human cadaver heads to document the stiffness of the temporo-parietal region and enhance fracture force data for this area. Each test involved sufficient energy to induce fractures, with impacts specifically targeting the temporo-parietal region. The experiments were performed using a drop tower, and the specimens ranged in age from 19 to 90 years, comprising 19 females and 12 males.

In addition, it is important to note that in this study the authors report the same height and velocity for all tests performed with circular and rectangular impactors. However, it is quite possible that impacts were made at different heights and therefore at different velocities. Therefore, these velocities were excluded from the analyses.

IMPACTOR AREAS

The tests were performed using two impactors:

- Flat circular plate with a contact surface diameter of 2.54 cm and an edge radius of 2 mm, which had an area of 5 cm^2^(according to the authors)
- Flat rectangular plate approximately 5 cm by 10 cm, which according to the authors had an area of 52 cm^2^.

FRACTURE CLASSIFICATION

Authors did not use specific criteria to characterize the fractures they observed. However, they reported applying sufficient energy to produce significant fractures in the specimens. Therefore, all specimens were fracture.

**MCINTOSH, ET AL.** [**^24^**](#_ENREF_24)

SAMPLE

In 1993, [McIntosh, et al. ^24^](#_ENREF_24) conducted a study involving 17 unembalmed cadavers, in which they performed a total of 21 impact tests. For our analysis, we selected only 10 tests conducted on 10 specific cadavers (8603, 8604, 9202, 9204, 8539, 9209, 9211, 9213, 9208, and 9221), since the remaining 11 tests were performed under padded conditions. The selected cadavers were subjected to low-velocity, direct occipital and lateral head impacts to more precisely define the biomechanical responses of the head, examine the relationships between impact variables, head responses, and injuries, and propose potential improvements for head injury criteria and tolerance levels. Among these 10 cadavers, seven were male and three were female, with ages ranging from 21 to 77 years. The following tests were discarded:8601D, 8602D, 8542D, 8543D, 8540D, 8541D, 8548D, 8549D, 9215D, 9217D and 9218D.

IMPACTOR AREA

The impactor used was a wide and flat circular surface with a diameter of 15 cm. The area was calculated as a circle. The area is approximately:

$$A=\pi{*r}^{2}= \pi*\left( \frac{15}{2} \right)^{2}={176.71 cm}^{2}$$

FRACTURE CLASSIFICATION

The 1990 Abbreviated Injury Scale (AIS) version was used to describe the injuries produced in these studies. According to this scale, the authors describe four levels of injury: no fracture, linear fracture, multiple linear fractures, and basal fracture. The linear and multiple linear fractures corresponded to our description of linear fractures. The basal fracture was also interpreted as a linear fracture based on the figures presented in the paper, which state that a common skull fracture pattern detected in side impacts is "the linear fracture running in the middle cranial fossa and extending to the carotid canal” [McIntosh, et al. ^24^](#_ENREF_24).

**YOGANANDAN, ET AL.** [**^4^**](#_ENREF_4)

SAMPLE

In 1995, [Yoganandan, et al. ^4^](#_ENREF_4) conducted experiments on unembalmed human heads to determine biomechanical responses under quasi-static and dynamic loading conditions. The study utilized a total of 12 cadavers. However, only specimens numbered 7 through 12 were subjected to dynamic conditions. These specimens included four females and two males, aged between 50 and 78 years. Specimens 1 through 6 were excluded from the database. These specimens were impacted under quasi-static conditions.

IMPACTOR AREA

The impactor employed was a hemisphere anvil with a 4.8 cm radius. The area was calculated as a circle, since only the base of the hemisphere contacts the surface. The area is approximately:

$$A=\pi{*r}^{2}= \pi{*(4.8)}^{2}=72.38 {cm}^{2}$$

FRACTURE CLASSIFICATION

The authors do not describe the fracture classification used. However, they mention different categories to describe them: linear, circular and multiple fractures. The description of linear fractures is compatible with our characterization criteria, but circular and multiple fractures are not. The criteria of multiple and circular fracture (specimens 8, 9, 10, 11, 12) were ruled out because of the ambiguity in associating them with the criteria employed, but the experimental subjects were included because they present fractures.

**VERSCHUEREN, ET AL.** [**^25^**](#_ENREF_25)

SAMPLE

In 2007, [Verschueren, et al. ^25^](#_ENREF_25) a new set-up for cadaver head impact testing was developed and validated. In this study, four embalmed human heads and four skulls underwent a total of 14 frontal impacts**.** For the database, we selected only the eight impacts conducted on the human heads, discarding those performed on skulls. However, head 4 was subsequently excluded from analysis, as only loading velocity data was provided for it.

IMPACTOR AREA

The impactor used was a flat-surfaced cylindrical with a 7 cm diameter. The area was calculated as the circular base of the cylinder, as the impact was made with the flat base of the cylinder. The area is approximately:

$$A=\pi*r^{2}= \pi*\left( \frac{7}{2} \right)^{2}={38.48 cm}^{2}$$

FRACTURE CLASSIFICATION

The authors used two categories, "linear" and "depressed," to describe the fractures observed in their tests. However, these terms were not explicitly defined. Despite this lack of definition, the categories closely match the characterization criteria we use.

**DELYE, ET AL.** [**^6^**](#_ENREF_6)

SAMPLE

In 2007, [Delye, et al. ^6^](#_ENREF_6) conducted a study with 18 unembalmed post-mortem human subjects to investigate whether an energy failure level applies to the skull fracture mechanics in unembalmed post-mortem human heads under dynamic frontal loading conditions. The subjects' ages ranged from 62 to 95 years, including seven males and eleven females. The specimens were impacted on the frontal bone, using the same double-pendulum methodology tested by [Verschueren, et al. ^25^](#_ENREF_25).

IMPACTOR AREA

The impactor used was the same as that employed by [Verschueren, et al. ^25^](#_ENREF_25).

FRACTURE CLASSIFICATION

Authors did not give much information about the fracture patterns. They mentioned that all specimens were eventually fractured, with linear fracture patterns typically in the low and intermediate velocity groups. The paper illustrates the type of fracture produced by low and medium velocity impacts in Figure 3. It is described in the results as follows: “The linear fracture originated at the point of impact and extended towards one of the orbital rims” [Delye, et al. ^6^](#_ENREF_6).

Figure 4, shows the type of fracture produced by a high velocity impact is also depicted. It is described in the results as follows: “The fracture pattern was more complex, although the linear fracture line towards the orbital rim was still visible in all cases” [Delye, et al. ^6^](#_ENREF_6). In accordance with this information, we decided to characterise all fractures produced as linear fractures.

**KROMAN, ET AL.** [**^26^**](#_ENREF_26)

SAMPLE

In 2011, [Kroman, et al. ^26^](#_ENREF_26) conducted a study with the objective of re-examining Gurdjian's theories of fracture propagation and re-evaluating the relationship between impact site and fracture pattern. To this end, five cadaver heads, comprising two females and three males with an age range of 61–89 years, were included in the study.

The head specimens (1–4) were stabilized underneath by a wooden support board that was scored with a saw in a collinear manner along the direction of impact. This ensured that the beam would fail completely well before the skulls began to fracture. In other words, the wooden support board served to maintain the skull's position during impact but failed immediately at the slightest increase in pressure. The test with head specimen 5 was excluded from the database because the authors modified the impact conditions to a semi-rigid boundary. The impact resulted in fractures in both parietal bones.

IMPACTOR AREA

The authors did not provide information on the impactor used, so this study was excluded from the fracture analyses.

FRACTURE CLASSIFICATION

Authors did not specify the criteria used to classify fractures; however, they described radial and concentric fractures, concepts that align closely with our own. Additionally, a small fracture with a stellate pattern, limited to the outer table, was documented in specimen 3. This fracture is compatible with our description of a notch, as the authors noted that despite its stellate morphology, it was a minor fracture confined to the outer table.

**FENTON, ET AL. ^[5](#_ENREF_5)^ & ISA ET AL.^[12](#_ENREF_12)^**

From 2015 to 2021, [Fenton, et al. ^5^](#_ENREF_5) developed a project aimed at providing essential data to accurately interpret blunt cranial trauma. This project involved impact experiments on adult human cadaver heads to generate baseline documentation of the effects of various forensically relevant variables on cranial fracture initiation, propagation, and patterning. In order to develop these objectives 39 isolated, fresh human cadaver heads were obtained, and several subprojects were performed.

**1) Energy, Implement, and Impact Number Effects on Cranial Fracture**

In order to investigate the effects of impactors shape, input energy, and impact number on cranial fractures, various studies were carried out. Two of these studies aimed to perform free-motion single impacts at a baseline or relatively "low" energy and free-motion single impacts at a relatively "high" energy. Both experiments led to subsequent publications. The data from the "low" energy experiments correspond to the project report by [Fenton, et al. ^5^](#_ENREF_5). Meanwhile, the data from the "high" energy experiments are derived from a later publication by [Isa, et al. ^12^](#_ENREF_12), which provides a defined fracture analysis criterion.

SAMPLE FOR “LOW” ENERGY EXPERIMENTS

In the "low" energy experiments, 12 human heads were utilized, and impacts were directed at the low mid-parietal region, superior to the squamosal suture. The study employed a pneumatic piston to perform the free-motion impacts. These experiments are called "free motion" impacts because the trolley on which the head was placed was free to move away from the impactor after the blow, thus allowing for rotation of the head with the neck and separation of the head away from the impactor after contact.

IMPACTOR AREAS FOR “LOW” ENERGY EXPERIMENTS

Three different shaped aluminium impactors were employed:

A small focal surface measuring 2.85 cm in diameter and weighing 6.27 kg. The area was calculated as a circle. The area is approximately:

$$A=\pi{*r}^{2}= \pi*\left( \frac{2.85}{2} \right)^{2}=6.45 {cm}^{2}$$

A broad curved surface, a horizontally oriented cylinder 6.35 cm in diameter and 6.35 cm long, weighing 6.31 kg. The area was calculated using the circular cross-section of the cylinder. The area is approximately:

$$A=2*\pi*h=2*\left( 3.1416 \right)*\left( 3.175 \right)*\left( 6.35 \right)= {126.72 cm}^{2}$$

A broad flat surface 7.62 cm in diameter and weighing 6.30 kg. The area was calculated as a circle. The area is approximately:

$$A=\pi{*r}^{2}= \pi*\left( \frac{7.62}{2} \right)^{2}=45.60 {cm}^{2}$$

FRACTURE CLASSIFICATION FOR “LOW” ENERGY EXPERIMENTS


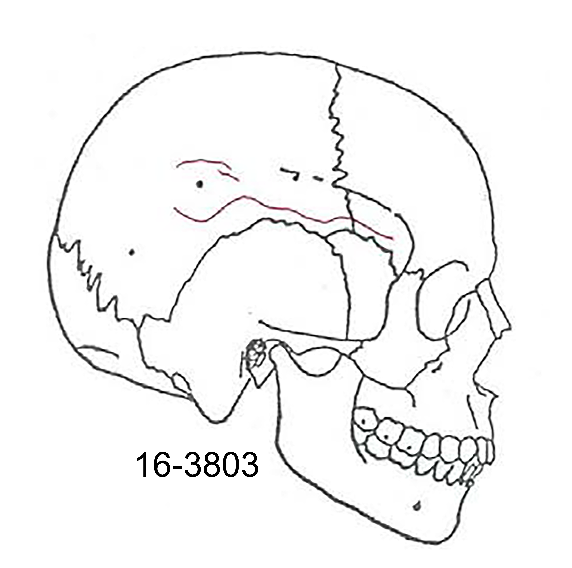
The authors described the lesions generated as linear, depressed, concentric, diastatic, or semicircular. This fracture criterion is similar to the one we used. However, in our case, we categorised the semicircular fractures as linear fractures based on the definition provided by [Isa, et al. ^12^](#_ENREF_12), as well as on observation of the fracture diagrams provided for the 16 – 3803 case (Supp. Fig. 1). Additionally, we introduced the criterion of comminution following our fracture criteria. This comminution criterion was employed by [Isa, et al. ^12^](#_ENREF_12) but not by [Fenton, et al. ^5^](#_ENREF_5). The low energy test impacts where we interpreted comminution to be present were: 16-3779, 17-0006 and 16-3801.

**Figure S9.** Fracture produced in skull number 16-3803 and characterised as semicircular following [Isa, et al. ^12^](#_ENREF_12). Figure from [Fenton, et al. ^5^](#_ENREF_5).

SAMPLE FOR “HIGH” ENERGY EXPERIMENTS

For the “high” energy experiments, the methodology and the number of specimens employed were identical to the corresponding “low” energy experiments. The impact energy, however, was 1.5 times higher.

IMPACTOR AREAS FOR “LOW” ENERGY EXPERIMENTS

The impactors used were the same as those used in the low-energy experiments. However, the weight was increased for each impactor.

- The small focal surface of 6.45 cm^2^ area increased its weight from 6.27 to 9.41 kg.
- The broad curved surface of 126.41 cm^2^ area increased its weight from 6.31 to 9.61 kg.
- The broad flat surface of 45.60 cm^2^ area increased its weight from 6.30 to 9.60 kg.

FRACTURE CLASSIFICATION FOR “LOW” ENERGY EXPERIMENTS

In the “high” energy experiments, Isa and co-workers provide a criterion used to classify the different injuries:

- Radial crack: Fracture that originates from within the zone of contact and propagates outward from the impact site.
- Remote linear fracture: Linear fracture that originates at a location other than the impact site.
- Cone crack: A conoidal fracture resulting from an object impacting or passing through a brittle material. It starts out as a circular shaped origin (ring crack) on the impact surface, then propagates outward and through the structure.
- Circumferential crack: Circular, semi-circular, or arc-shaped fracture surrounding an impact site that forms due to inward bending.
- Concentric crack: Two or more semi-circular fractures sharing a common centre.
- Depression: Bone is displaced endocranially.
- Comminution: Bone is broken up into smaller pieces


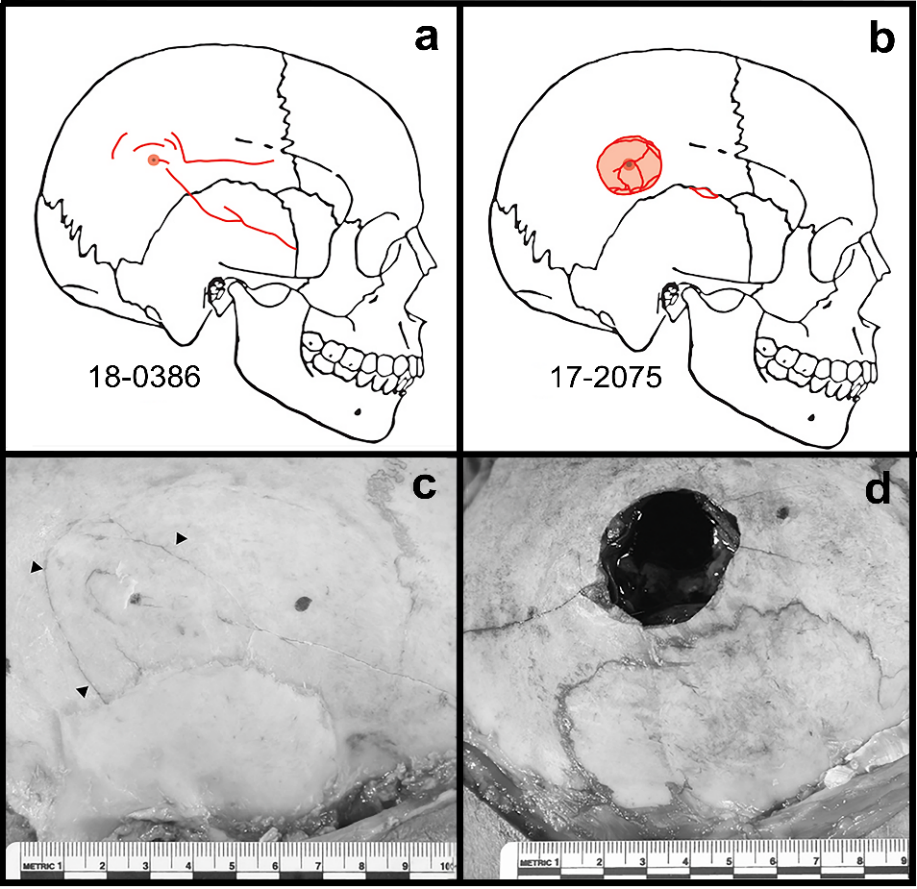
Radial crack, concentric crack, depression, comminution, and remote linear fracture are terms compatible with our criteria. Circumferential crack was interpreted as a linear fracture based on the definition provided by [Isa, et al. ^12^](#_ENREF_12), as well as on observation of the fracture diagrams provided for the 17 – 2071, 18 – 0386, 17 – 2081, 18 – 0364, 17 – 2118, 17 – 2132, 17 – 2095 and 18 – 0300 cases. (Supp. Fig. 2) Cone cracks are associated with depression or penetrating fractures because they occur when an object impacts the skull with sufficient force to generate a depressed or penetrating fracture. This study shows that cone cracks are indeed associated with these types of fractures. Therefore, in our analysis, cone cracks were categorized as either depressed or penetrating fractures, depending on the fracture description for the cases: 17 – 2075 and 18 – 2082 (Supp. Fig. 2). In addition, we also interpret that there are comminuted fractures in the cases: 17-2075, 18-2359 and 17-2095.

**Figure S10.** a) Fracture produced in skull number 18-0386 and characterised as a circumferential crack following [Isa, et al. ^12^](#_ENREF_12). b) Fracture produced in skull number 17-2075 and characterised as a cone crack following [Isa, et al. ^12^](#_ENREF_12). c) Example of circumferential crack. d) Example of cone crack. Figures from [Isa, et al. ^12^](#_ENREF_12).

**2) Constraint Condition Effects on Cranial Fracture**

In order to assess whether constraint conditions influenced the initiation of cranial fractures at or peripheral to the POI, single impacts were performed on an additional 22 heads. Two different methodologies were employed to conduct these impacts: A and B.

1. SAMPLE

Five constrained impacts were performed using a gravity drop impact system. Specimens were positioned for impact within a rigid medium made of plaster of Paris. The centre parietal area of the specimens was impacted. Additionally, data from seven other specimens previously captured in a pilot study were included. In total, data were obtained from 12 individuals.

A) IMPACTOR AREA

The authors used four different impactors:

A spherical impactor with a 2.54 cm diameter. The area was calculated as a circle. The area is approximately:

$$A=\pi{*r}^{2}= \pi*\left( 2.54 \right)^{2}=20.27 {cm}^{2}$$

A hemispherical impactor with a 5.08 cm diameter. The area was calculated as a circle. The area is approximately:

$$A=\pi{*r}^{2}= \pi*\left( \frac{5.08}{2} \right)^{2}=20.27 {cm}^{2}$$

A square flat impactor with a 2.54 cm diameter. The area was calculated as a square. The area is approximately:

$$A={side}^{2}= {2.54}^{2}=6.45 {cm}^{2}$$

A flat impactor with a 7.62 cm diameter. The area was calculated as a circle. The area is approximately:

$$A=\pi{*r}^{2}= \pi*\left( \frac{7.62}{2} \right)^{2}=45.60 {cm}^{2}$$

A) FRACTURE CLASSIFICATION


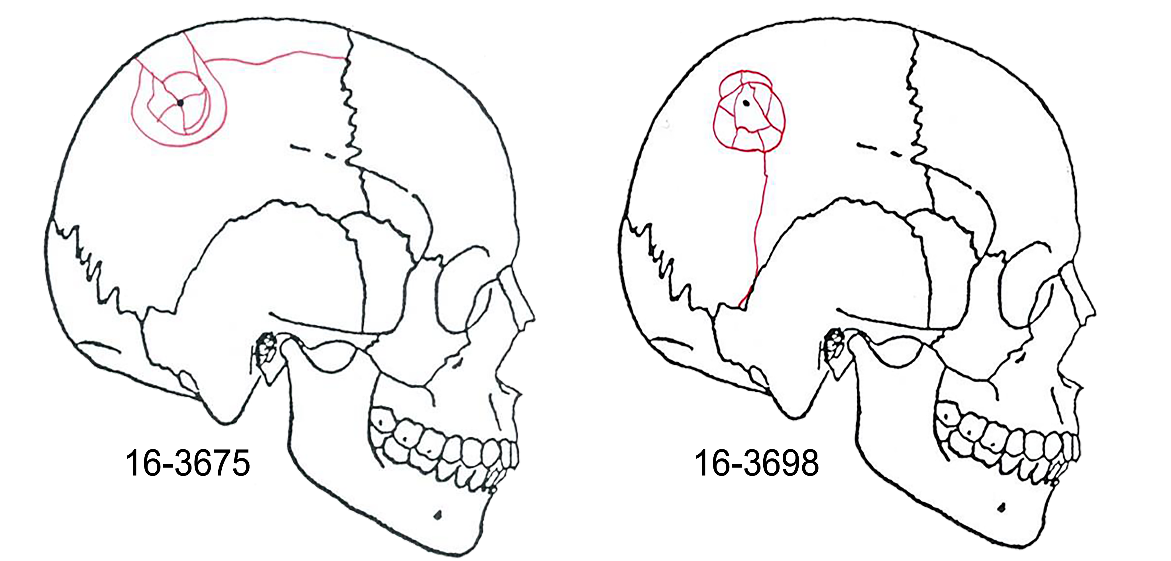
çThe fracture criterion is the same as that used in low-energy experiments. Additionally, we introduced the criterion of comminution following our fracture criteria. This criterion was employed by [Isa, et al. ^12^](#_ENREF_12) but not by [Fenton, et al. ^5^](#_ENREF_5). Based on this, we interpreted there to be comminuted fractures in: 16-3675, 14-2111, 16-3684, 16-3698, and 14-1792 (Supp. Fig 3).

**Figure S11.** Fractures produced in skulls 16-3675 and 16 3698. Note how the bone was fragmented into more than two pieces. Figures from [Isa, et al. ^12^](#_ENREF_12) and [Fenton, et al. ^5^](#_ENREF_5).

1. SAMPLE

Ten free-motion impacts were performed using the pneumatic system. The specimens were impacted once at the centre parietal. The specimen 18-3956 was excluded because no impact data were reported, it was only described in the study.

B) IMPACTOR AREA

The specimens were impacted using three of the same impactors from the constrained impacts:

- A square flat impactor with 6.45 cm^2^ area.
- A hemispherical impactor with 20.27 cm^2^ area.
- A flat impactor with 45.60 cm^2^ area.

B) FRACTURE CLASSIFICATION

The fracture criterion is the same that was used in the low-energy experiments. We interpreted there to be comminuted fractures in: 14-1822, 18-1092, 18-1101, 18-1083, 18-2261 and 18-3930.

**REFERENCES**

1 Hodgson, V. R., Brinn, J., Thomas, L. & Greenberg, S. Fracture behavior of the skull frontal bone against cylindrical surfaces. Report No. 700909, (SAE International, 1970). <https://doi.org/10.4271/700909>

2 Hodgson, V. R. & Thomas, L. Breaking strength of the human skull vs. impact surface curvature. Report No. FH-11-7609, 62 (Wayne State University, School of Medicine, Department of Neurosurgery, Detroit, MI, USA, 1971).

3 Hodgson, V. R. & Thomas, L. M. Breaking strength of the human skull vs impact surface curvature. Report No. DOT-HS-146-2-230, 194 (Wayne State University, School of Medicine, Department of Neurosurgery, Detroit, MI, USA, 1973).

4 Yoganandan, N. *et al.* Biomechanics of skull fracture. *J. Neurotrauma* **12** (1995). <https://doi.org/10.1089/neu.1995.12.659>

5 Fenton, T. W., Haut, R. C. & Wei, F. Building a science of adult cranial fracture. (Michigan State University, 2015).

6 Delye, H. *et al.* Biomechanics of frontal skull fracture. *J. Neurotrauma* **24**, 1576-1586 (2007). <https://doi.org/10.1089/neu.2007.0283>

7 Schneider, D. C. & Nahum, A. M. Impact studies of facial bones and skull. Report No. 720965, (SAE International, 1972). <https://doi.org/10.4271/720965>

8 Nahum, A. M., Gatts, J. D., Gadd, C. W. & Danforth, J. Impact tolerance of the skull and face. Report No. 680785, (SAE International, 1968). <https://doi.org/10.4271/680785>

9 Palomo Rando, J., Ramos Medina, V., Palomo Gómez, I., Lopez Calvo, A. & Santos Amaya, I. Patología forense y neurología asociada de los traumatismos craneoencefálicos. Estudio práctico. *Cuad. Med. Forense* **14**, 87-118 (2008).

10 Wedel, V. L. & Galloway, A. *Broken bones: anthropological analysis of blunt force trauma*. (Charles C. Thomas, 2014).

11 Gurdjian, E., Webster, J. E. & Lissner, H. The mechanism of skull fracture. *Radiology* **54**, 313-339 (1950). <https://doi.org/10.1148/54.3.313>

12 Isa, M. I. *et al.* Effects of input energy and impactor shape on cranial fracture patterns. *Forensic Sci. Int.* **352**, 111859 (2023). <https://doi.org/10.1016/j.forsciint.2023.111859>

13 Gurdjian, E. S. *Impact head injury: mechanistic, clinical, and preventive correlations*. (Charles C. Thomas, 1975).

14 Samandouras, G. *The neurosurgeon's handbook*. (Oxford University Press, 2010).

15 Vakil, M. T. & Singh, A. K. A review of penetrating brain trauma: epidemiology, pathophysiology, imaging assessment, complications, and treatment. *Emerg. Radiol.* **24**, 301-309 (2017). <https://doi.org/10.1007/s10140-016-1477-z>

16 Betz, P., Stiefel, D., Hausmann, R. & Eisenmenger, W. Fractures at the base of the skull in gunshots to the head. *Forensic Sci. Int.* **86**, 155-161 (1997). <https://doi.org/10.1016/S0379-0738(97)02121-X>

17 Gurdjian, E. S., Webster, J. E. & Lissner, H. R. Studies of skull fracture with particular reference to engineering factors. *Am. J. Sur.* **78**, 736-742 (1949). <https://doi.org/10.1016/0002-9610(49)90315-3>

18 Yoganandan, N. & Pintar, F. A. Biomechanics of temporo-parietal skull fracture. *Clin. Biomech* **19**, 225-239 (2004). <https://doi.org/10.1016/j.clinbiomech.2003.12.014>

19 Gurdjian, E. S., Webstef, J. & Lissner, H. R. Observations on prediction of fracture site in head injury. *Radiology* **60**, 226-235 (1953). <https://doi.org/https://doi.org/10.1148/60.2.226>

20 Stalnaker, R., Melvin, J., Nusholtz, G., Alem, N. & Benson, J. Head impact response. Report No. 770921, (SAE International, 1977). <https://doi.org/10.4271/770921>

21 Got, C., Patel, A., Fayon, A., Tarriere, C. & Walfisch, G. Results of experimental head impacts on cadavers: the various data obtained and their relations to some measured physical parameters. Report No. 780887, (SAE International, 1978). <https://doi.org/10.4271/780887>

22 Allsop, D. L., Warner, C. Y., Wille, M. G., Schneider, D. C. & Nahum, A. M. Facial impact response—a comparison of the Hybrid III dummy and human cadaver. Report No. 881719, (SAE International, 1988). <https://doi.org/10.4271/881719>

23 Allsop, D. L., Perl, T. R. & Warner, C. Y. Force/deflection and fracture characteristics of the temporo-parietal region of the human head. Report No. 912907, (SAE International, 1991). <https://doi.org/10.4271/912907>

24 McIntosh, A., Kallieris, D., Mattern, R. & Miltner, E. Head and neck injury resulting from low velocity direct impact. Report No. 933112, (SAE International, 1993). <https://doi.org/10.4271/933112>

25 Verschueren, P. *et al.* A new test set-up for skull fracture characterisation. *J. Biomech.* **40**, 3389-3396 (2007). <https://doi.org/10.1016/j.jbiomech.2007.05.018>

26 Kroman, A., Kress, T. & Porta, D. Fracture propagation in the human cranium: a re-testing of popular theories. *Clin. Anat.* **24**, 309-318 (2011). <https://doi.org/10.1002/ca.21129>
